# Elongated Structure of the Outer-Membrane Activator of Peptidoglycan Synthesis LpoA: Implications for PBP1A Stimulation

**DOI:** 10.1016/j.str.2014.04.017

**Published:** 2014-07-08

**Authors:** Nicolas L. Jean, Catherine M. Bougault, Adam Lodge, Adeline Derouaux, Gilles Callens, Alexander J.F. Egan, Isabel Ayala, Richard J. Lewis, Waldemar Vollmer, Jean-Pierre Simorre

**Affiliations:** 1 University Grenoble Alpes, Institut de Biologie Structurale, F-38027 Grenoble, France; 2 CEA, DSV, Institut de Biologie Structurale, F-38027 Grenoble, France; 3 CNRS, Institut de Biologie Structurale, F-38027 Grenoble, France; 4 The Centre for Bacterial Cell Biology, Institute for Cell and Molecular Biosciences, Newcastle University, Richardson Road, Newcastle upon Tyne NE2 4AX, UK; 5 Institute for Cell and Molecular Biosciences, Newcastle University, Framlington Place, Newcastle upon Tyne NE2 4HH, UK

## Abstract

The bacterial cell envelope contains the stress-bearing peptidoglycan layer, which is enlarged during cell growth and division by membrane-anchored synthases guided by cytoskeletal elements. In *Escherichia coli*, the major peptidoglycan synthase PBP1A requires stimulation by the outer-membrane-anchored lipoprotein LpoA. Whereas the C-terminal domain of LpoA interacts with PBP1A to stimulate its peptide crosslinking activity, little is known about the role of the N-terminal domain. Herein we report its NMR structure, which adopts an all-α-helical fold comprising a series of helix-turn-helix tetratricopeptide-repeat (TPR)-like motifs. NMR spectroscopy of full-length LpoA revealed two extended flexible regions in the C-terminal domain and limited, if any, flexibility between the N- and C-terminal domains. Analytical ultracentrifugation and small-angle X-ray scattering results are consistent with LpoA adopting an elongated shape, with dimensions sufficient to span from the outer membrane through the periplasm to interact with the peptidoglycan synthase PBP1A.

## Highlights

• LpoA’s N-terminal domain features an all-α-helical fold similar to TPR domains
• The C-terminal domain of *E. coli* LpoA contains two extensive flexible regions
• Full-length LpoA adopts an elongated structure with low interdomain flexibility
• LpoA can span the periplasm to stimulate the peptidoglycan synthase PBP1A

## Graphical Abstract

**Figure:**
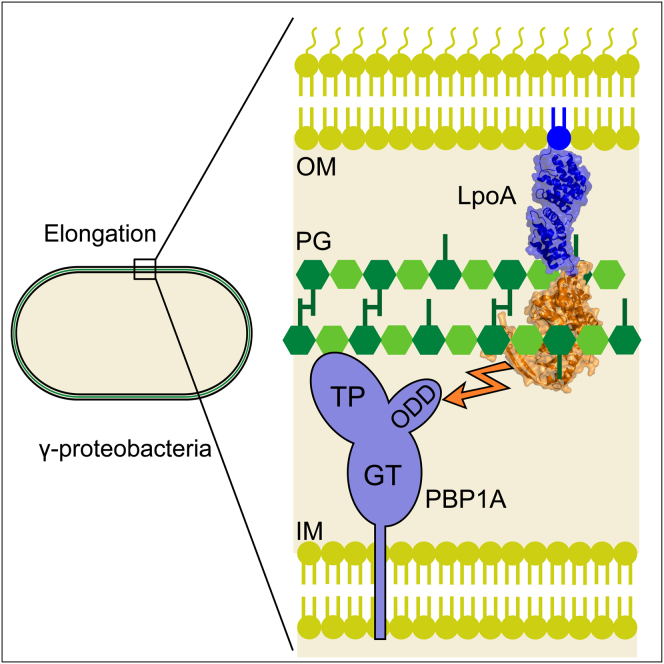

## Teaser

Jean et al. determine the NMR structure of LpoA N-terminal domain and identify two unique flexible regions in the C-terminal domain. SAXS data suggest little flexibility between the two domains leading to an elongated shape for the full-length protein, required in the activation of peptidoglycan synthase PBP1A.

## Introduction

Most bacteria surround their cytoplasmic membrane with a peptidoglycan (PG) sacculus, which maintains cell shape and protects the cell from lysis due to turgor. The PG sacculus is an elastic, net-like molecule composed of glycan chains that are connected by short peptides (Vollmer et al., 2008). Gram-negative bacteria, such as *Escherichia coli*, have a thin, mainly single-layered PG and an outer membrane containing lipopolysaccharide.

Growing and dividing cells polymerize the PG precursor lipid II and insert it into the existing PG layer by the combined actions of PG synthases and hydrolases, which presumably form dynamic, membrane-attached multienzyme complexes (Vollmer et al., 2008). *E. coli* has six cytoplasmic-membrane-anchored PG synthases. PBP1A and PBP1B are the major (and semiredundant) bifunctional glycosyltransferase-transpeptidases, with well characterized in vitro activities (Banzhaf et al., 2012, Bertsche et al., 2005, Born et al., 2006). PBP2 and PBP3 are transpeptidases that are essential for cell elongation and division, respectively.

PG synthesis and hydrolysis are regulated from inside the cell by cytoskeletal elements (Typas et al., 2012). In *E. coli* and presumably in other Gram-negative bacteria, PG synthesis is also regulated from outside the sacculus. PBP1A and PBP1B both require cognate outer-membrane-anchored lipoproteins (LpoA and LpoB, respectively) for function (Paradis-Bleau et al., 2010, Typas et al., 2010). LpoA and LpoB are unrelated in amino acid sequence, but both attach to the inner leaflet of the outer membrane by an N-terminal lipid modification. The C-terminal domain of LpoA interacts with PBP1A to stimulate its transpeptidase activity by an as yet unknown mechanism, whereas LpoB interacts with the noncatalytic UB2H domain of PBP1B to stimulate its glycosyltransferase and transpeptidase activities. In the cell, the Lpo proteins must reach through pores in the PG net to interact with their cognate PG synthase. Therefore, it has been proposed that the Lpo-mediated activation of PBPs occurs in response to the properties of the pores in the elastic PG layer to adjust the rate of PG growth with the rate of cell growth (Typas et al., 2010, Typas et al., 2012). Supporting this model, a previous study showed that both outer-membrane-anchored Lpo proteins could be crosslinked to their cognate cytoplasmic membrane-anchored PBP in the cell (Typas et al., 2010). However, in the absence of structural data for full-length LpoA and LpoB, it has remained unclear whether both proteins are long enough to span a distance of at least 110 Å from the outer membrane through the PG layer to interact with their cognate PBP.

The crystal structure of the C-terminal domain of *Haemophilus influenzae* LpoA (LpoA^C^, residues 257–573) reveals two subdomains that adopt a fold commonly found in periplasmic substrate-binding proteins (Vijayalakshmi et al., 2008). *E. coli* LpoA^C^ has two additional stretches of 67 and 39 amino acids, respectively, that are missing in the *H. influenzae* ortholog. The role of the N-terminal domain (LpoA^N^) is not clear, as it does not interact with PBP1A (Typas et al., 2010).

In this work, we present the structure of LpoA^N^ determined by NMR spectroscopy. LpoA^N^ is almost completely made of α-helices that form helix-turn-helix motifs similar to tetratricopeptide repeat (TPR) motifs, which are commonly used as a scaffold underpinning protein/protein complexes. We also provide evidence from NMR spectroscopy that LpoA^C^ contains flexible (unstructured) regions and that the two domains of LpoA are connected by a rather rigid linker. Further biophysical characterization by analytical ultracentrifugation (AUC) and small-angle X-ray scattering (SAXS) showed that full-length LpoA has an elongated molecular shape, supporting the hypothesis that LpoA has the capacity to span the distance from the outer membrane to its cognate cytoplasmic-membrane-anchored PG synthase, PBP1A.

## Results

### The N-Terminal Domain of LpoA Adopts a TPR-Domain-like Fold

**Figure 1:**
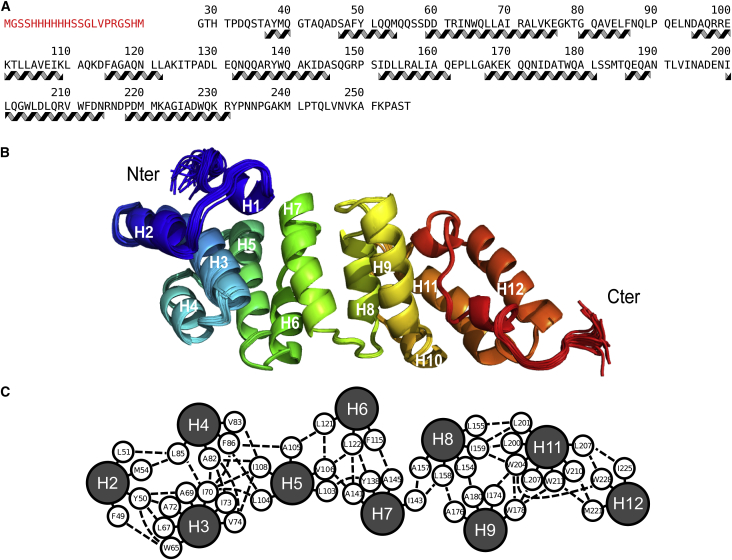
LpoA^N^ Has a TPR Domain-like Structure (A) Amino acid sequence of the LpoA^N^ construct used for structure determination and secondary structure elements (α helices). The residues of the oligohistidine tag are shown in red. (B) Cartoon representation of the 20 lowest-energy structures of LpoA^**N**^, as determined by NMR spectroscopy, emphasizing the spatial arrangement of the 12 α-helices, which are numbered from the N to C terminus. (C) LpoA^N^ is stabilized by numerous interhelical hydrophobic contacts. The ten α helices with more than four residues are shown as circles and labeled according to the description in (B). Interhelical hydrophobic contacts are represented with dashed lines, and short plain lines connect each of the residues to its corresponding helix. See also Figure S1.

We purified the N-terminal domain of *E. coli* LpoA lacking its signal peptide sequence and outer-membrane lipid anchor (LpoA^N^, residues 28–256) fused to an N-terminal oligohistidine tag (Figure 1A and Figure S1A available online). ^1^H-^15^N heteronuclear single quantum coherence (^1^H-^15^N-HSQC) NMR spectra were recorded from ^13^C,^15^N-LpoA^N^ samples at different temperatures (5°C–50°C) and at different pH values (4.5–7.5), and showed that the global fold of the protein remained unchanged (Figure S1B). For structure determination, NMR spectra were recorded at 50°C and pH 4.5, and chemical shifts were assigned (Jean et al., 2014). The structure of LpoA^N^ (Figures 1B and 1C) was subsequently determined. Relevant structural and statistical data are reported in Table 1.

**Table 1:** Structural Statistics for the Ensemble of 20 NMR Structures of LpoA^N^

NMR Distance and Dihedral Constraints	Number/Parameter
**Distance Constraints**	

Total unambiguous NOE restraints	5,210
Intraresidue	1,826
Interresidue	3,384
Sequential (|*i* – *j*| = 1)	1,141
Medium-range (|*i* – *j*| ≤ 5)	1,225
Long-range (|*i* – *j*| > 5)	1,018
Total ambiguous NOE restraints	440

**Total Dihedral Angle Restraints**	426

Backbone Φ	213
Backbone Ψ	213

**Structure Calculation Statistics**^a^	

**Restraints Violations**	

Distance (>0.3 Å, >0.5 Å)	41.95, 4.5
Dihedral (>5°, >6°)	13, 0
Average pairwise root-mean-square deviation (Å)^b^	
Backbone atoms	0.44 ± 0.06
All heavy atoms	0.63 ± 0.05

**Ramachandran Analysis**^b^	

Residues in most favored regions (%)	85.2
Residues in additional allowed regions (%)	13.6
Residues in generously allowed regions (%)	1.0
Residues in disallowed regions (%)	0.2

a Pairwise deviations were calculated among 20 refined structures.

b These values were calculated on residues 28–256 (according to numbering in wild-type LpoA).

LpoA^N^ is made up of 12 α-helices (H1–H12) of variable length linked together through short turns or rigid loops (Figure 1B). Two additional short stretches of three residues at the C terminus also adopt a characteristic 3_10_-helix conformation (residues 236–238 and 246– 248). There are numerous interhelical hydrophobic contacts (Figure 1C) favored by the high percentage (24%) of long-chain hydrophobic amino acids and few additional side-chain hydrogen bonds, which explains the stability of the structure over wide pH and temperature ranges (Figure S1B).

LpoA^N^ has a striking structural similarity to protein domains formed by TPR motifs, as revealed by DALI (Holm and Rosenström, 2010), consistent with the repetition of helix-turn-helix motifs found in these proteins and LpoA^N^. The highest score (Z = 9.3) was obtained with a 170-residue stretch of the G protein signaling regulator 2 (Protein Data Bank [PDB] code 3SF4), but both structures had a relatively high root-mean-square deviation (RMSD) value of 5.7 Å. Searching the LpoA^N^ sequence for canonical TPR repeats (Zeytuni and Zarivach, 2012), TPRpred (Biegert et al., 2006) recognized a TPR sequence pattern only in the I63-A96 fragment comprising H3 and H4, although H4 is shorter than the typical α-helices in TPR motifs. H11/H12 shows a substantial structural similarity to TPR motifs, but was not classified as a TPR motif by TPRpred because long-chain hydrophobic residues replace the conserved alanine residues at positions 8, 20, and 27. The similarity between LpoA^N^ and TPR domains thus seems limited to the presence of a series of helix-turn-helix motifs with short hydrogen-bonded turns between the two helices (H3/H4, H5/H6, H8/H9, and H11/H12) rather than to the presence of canonical TPR repeats with their characteristic hydrophobic contacts. As in most TPR domains, the individual helices in LpoA^N^ are organized into a superhelical structure; however, the curvature of this structure is too small to generate the concave and convex surfaces often seen in TPR domains. Instead, LpoA^N^ shows an overall prolate spheroid shape with a width of ∼30 Å and a height of ∼70 Å. The protein surface contains three grooves. Two of these grooves localize between the central helices H7 and H8, and the third localizes between H3 and H5. Interestingly, these grooves contain highly conserved residues and are therefore potential interaction sites with proteins or other ligands (Figure S1C).

### Full-Length LpoA Has Flexible Regions in the C-Terminal Domain

We next sought to determine the overall molecular shape for full-length LpoA and the relative orientation of the two domains. The N-terminally oligohistidine-tagged protein (Figure S1A) was mostly monomeric (∼85%) and contained ∼10% dimers. This ratio was similar for all three protein concentrations (6, 2, and 0.2 mg/ml) used in AUC experiments, suggesting that monomers and dimers are not in dynamic exchange. The f/f_min_ ratio of 1.5 and hydrodynamic radius of 4.1 nm, determined from the sedimentation coefficient extrapolated to infinite dilution, s_0_ = 4.16 S, are characteristic of an elongated molecular shape and/or a flexible molecule.

**Figure 2:**
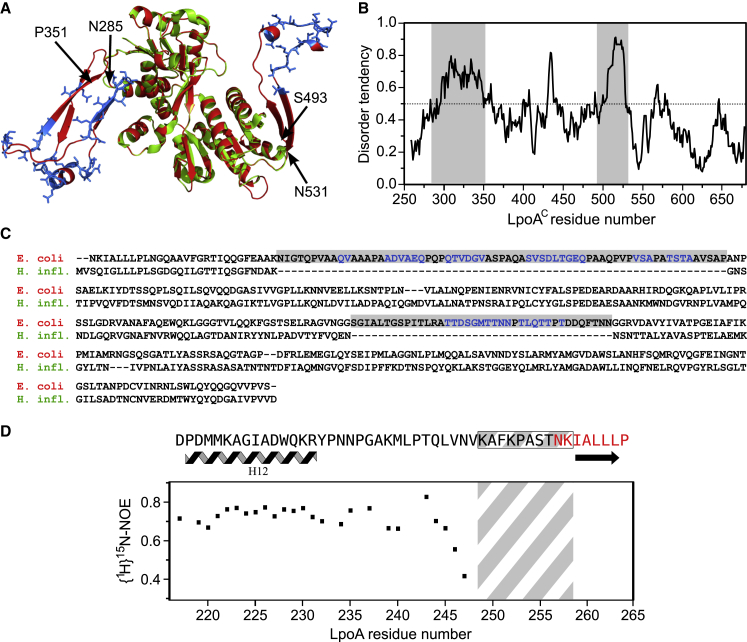
Comparison of LpoA^C^ from *E. coli* and *H. influenzae* (A) Superimposition of the X-ray structure of *H. influenzae* LpoA^C^ (green, PDB code 3CKM) and the structure of *E. coli* LpoA^C^ predicted by PHYRE (red). Regions 1 (residues N285–P351) and 2 (residues S493–N531) are present in LpoA^C^ from *E. coli*, but not from *H. influenzae*. Flexible residues for which backbone resonances have been assigned by NMR spectroscopy are sketched as blue sticks. (B) Disorder in *E. coli* LpoA^C^ predicted by IUPred. The two main regions absent from the *H. influenzae* LpoA sequence (in gray) are predicted as being mostly unstructured by IUPred (score > 0.5). (C) Sequence alignment of *H. influenzae* and *E. coli* LpoA^C^, where the two *E. coli* inserts are highlighted in gray. NMR-assigned residues are shown in blue. (D) The linker between LpoA^N^ and LpoA^C^ (in gray and white hashes) starts at K249 and ends at K258. The criteria used to define this linker included the structuring of the N-terminal domain as quantified by the {^1^H}^15^N-NOE measured in LpoA^N^ (black) and the definition of the first secondary-structure element in the C-terminal domain (red) of LpoA as modeled by PHYRE. See also Figure S2.

Full-length LpoA (see Figure 2 for LpoA^C^) has a molecular weight of >70 kDa and therefore is too large for structure determination by NMR spectroscopy. Nonetheless, a ^1^H-^15^N band-selective excitation short-transient transverse optimized spectroscopy (^1^H-^15^N-BEST-TROSY) NMR spectrum of full-length ^13^C,^15^N-LpoA showed ∼100 intense and tightly dispersed signals (Figure S2A). The signals seen in LpoA^N^ were all absent in this spectrum, suggesting that the molecular tumbling of the large anisotropic LpoA molecule prevents the NMR observation of structured (or nonflexible) regions and that only residues in disordered (or flexible) parts of the molecule could be seen. LpoA^N^ is well structured and therefore most of the signals obtained from full-length LpoA should come from disordered regions in the C-terminal domain. To investigate this possibility, we assigned the detected ^1^H, ^13^C, and ^15^N resonances of a full-length ^13^C,^15^N-LpoA sample. HNCACB and BEST-TROSY-(H)N(COCA)NH experiments unambiguously identified stretches of contiguous residues that all reside within the two regions of the *E. coli* LpoA sequence that are absent in *H. influenzae* LpoA (Figure 2). These assignments included backbone resonances from 30 residues between N285 and P351 (region 1) and from 16 residues between S493 and N531 (region 2; Figure S2B). Amino acid types were obtained from Cα and Cβ carbon chemical shifts for 36 additional resonances (leaving only eight resonances completely unassigned) and agreed with the expected unassigned amino acids in the two considered regions. In line with these data, regions 1 and 2 were predicted to be unfolded by IUPred (http://iupred.enzim.hu/) with scores higher than 0.5 (Figure 2B).

We subsequently built a structural model of *E. coli* LpoA^C^ using PHYRE (Kelley and Sternberg, 2009) with the *H. influenzae* LpoA^C^ structure as a template (Vijayalakshmi et al., 2008; PDB code 3CKM). The superimposition of the *H. influenzae* structure and the *E. coli* model (Figure 2A) emphasizes the presence of extended loops in the flexible regions 1 and 2. To evaluate this model in the light of experimental data, we analyzed chemical shifts from assigned residues in regions 1 and 2, and observed marked dynamics and low secondary-structure propensities at these positions (see Supplemental Experimental Procedures). As a result, region 1 might be more flexible and/or disordered than suggested by the model. In addition to the established loop in region 2, the PHYRE-predicted β sheet (formed by S493-G499 and D525-N531) could not be confirmed by NMR spectroscopy due to incomplete sequence-specific assignments.

### LpoA Has a Limited Interdomain Flexibility

If there is a highly flexible linker between the N- and C-terminal domains, full-length ^13^C,^15^N-LpoA should give rise to NMR signals corresponding to a sum of the signals observed for the separate domains. However, characteristic LpoA^N^ resonances remained undetected in this sample after an increase of the signal-to-noise ratio by a factor of 2. Only the virtually complete perdeuteration of full-length LpoA and the spectroscopic advantage of the TROSY effect allowed the detection of LpoA^N^ and LpoA^C^ amide resonances (Figure S2C). The structured regions of full-length LpoA, comprising most of the N- and C-terminal domains, thus behave as a highly anisotropic single structural entity without significant flexibility between the domains. This hypothesis was further confirmed by the similar heteronuclear multiple quantum coherence (HMQC) versus HSQC intensity ratios (Tugarinov et al., 2003) for ^1^H-^13^C correlations of alanine methyl groups in a U-[^2^H, ^12^C, ^15^N], Val-[^13^C^1^H_3_]^*pro-S*^, Ala-[^13^C^1^H_3_]-LpoA sample (Figure S2D), indicating a correlated rotational motion of the two domains. A strong, rather rigid interaction between LpoA^**N**^ and LpoA^**C**^ also agrees with the low disorder score of <0.38 calculated by IUPred for the linker region.

### Full-Length LpoA Has an Elongated Molecular Shape

**Figure 3:**
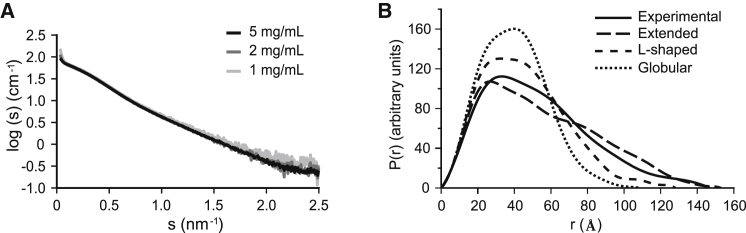
Full-Length LpoA Has an Extended Structure (A) SAXS curves of LpoA at concentrations of 1, 2, and 5 mg/ml. (B) Experimental distance distribution function, P(r), calculated from SAXS data collected on a 5 mg/ml ^15^N-LpoA sample (black) and theoretical P(r) function calculated for molecular models with three different, arbitrarily chosen orientations of the N- and C-terminal domains (....., globular model; ----, L-shaped model; – – –, extended model; see also Figure S3C). The theoretical R_g_ values extracted from these curves are 3.11 nm for the globular model, 3.58 nm for the L-shaped model, and 4.44 nm for the extended model. The experimental R_g_ value, 4.22 ± 0.01 nm, fits best to the extended model.

We used SAXS to determine the overall molecular shape of full-length LpoA (Figure 3). The estimated molecular weights of LpoA by SAXS (66.7 kDa, 64.9 kDa, and 68.2 kDa) were similar for each concentration tested (5, 2, and 1 mg/ml, respectively), showing that the scattering intensities recorded came mostly from the monomeric form of the protein and confirming the largely monomeric and monodisperse behavior of LpoA (Figure 3A). The distance distribution function P(r) was calculated from the SAXS data recorded at the highest protein concentration (Figure 3B) and yielded a radius of gyration (R_g_) of 4.22 ± 0.01 nm, which is similar to the R_g_ of 4.17 ± 0.02 nm calculated by the Guinier approximation (Figure S3A).

We next built hypothetical models of full-length LpoA with different relative orientations of the N- and C-terminal domains using the structure of LpoA^N^ and the previously built *E. coli* LpoA^C^ homology model (Figure S3C) (Svergun, 1992), and calculated their theoretical P(r) and R_g_ values. The elongated putative LpoA model had a theoretical R_g_ that was close to the experimental value (4.44 nm versus 4.22 nm), whereas the putative globular and L-shaped models had lower theoretical R_g_ values (3.11 nm and 3.58 nm, respectively). The observed P(r) is typical of an oblate shape and is similar to the curve calculated for the elongated model (Figure 3B). The calculated D_max_ (the longest distance between two points in the protein) of 146.8 Å is also consistent with an elongated arrangement of the two domains, as LpoA^N^ and LpoA^C^ have domain lengths of ∼70 Å and ∼60 Å, respectively. Thus, although the presence of extended flexible regions (regions 1 and 2) in the C-terminal domain (Figure S3B) prevented us from building a higher-resolution model of LpoA, the SAXS data are consistent with an elongated structure.

## Discussion

In this work, we determined the structure of the N-terminal domain of LpoA and showed that it adopts a TPR-like fold. Proteins containing TPR domains (or modules) are ubiquitous and are present in organisms belonging to all kingdoms of life (Zeytuni and Zarivach, 2012). It is generally accepted that TPR modules do not exhibit an enzymatic activity, but rather serve as interaction modules in multiprotein assemblies or for protein dimerization (Allan and Ratajczak, 2011). For example, the TPR domain of BamD, an essential component of the outer-membrane β-barrel assembly machinery in Gram-negative bacteria, interacts with the N-terminal, unstructured region of BamC (Kim et al., 2011, Sandoval et al., 2011).

The analysis of 63 unique LpoA^N^ sequences revealed two conserved patches on the protein surface, which could represent potential interaction sites (Figure S1C). PBP1A interacts with LpoA^C^, but not with LpoA^N^ (Typas et al., 2010), and LpoA and LpoA^N^ exist predominantly as monomers, suggesting that the TPR-like motifs do not promote self-interaction. Rather, LpoA^N^ may interact with one or more as yet unknown protein(s). Studies to identify the interaction partners of LpoA^N^ are currently under way.

Full-length LpoA yielded NMR signals that originate from two flexible regions in the C-terminal domain. Other species, such as *H. influenzae*, either lack these stretches or have different stretches at the same or a different position in the LpoA^C^ sequence (Vijayalakshmi et al., 2008). The functions of the two flexible regions in *E. coli* LpoA^C^ are currently unknown. It is possible that they fold into rigid structures under certain conditions, perhaps during the formation of new protein-protein interactions.

**Figure 4:**
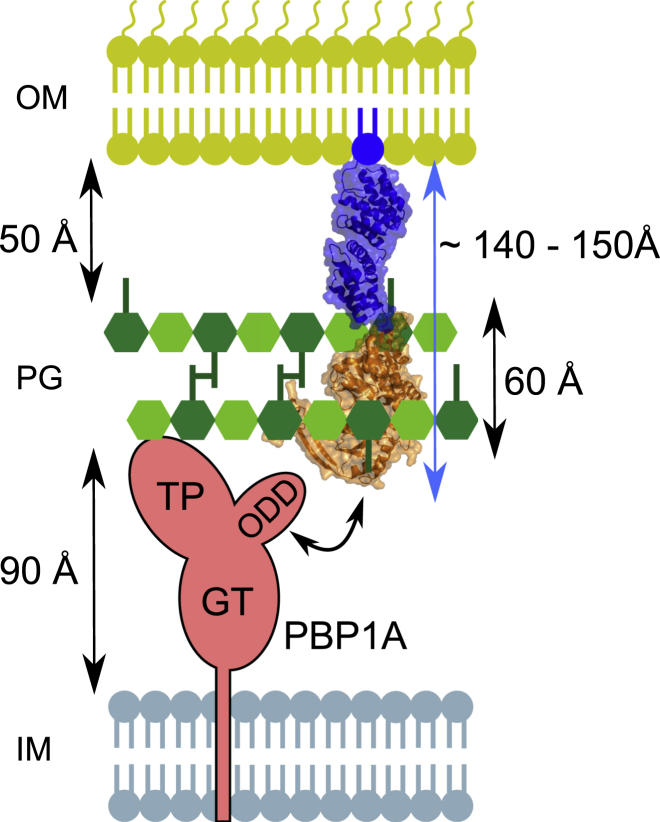
Schematic Representation of PBP1A Activation by LpoA The N-terminal domain of LpoA (blue) anchors to the outer membrane, whereas the C-terminal domain (orange) interacts with the outer-membrane PBP1A docking domain (Typas et al., 2010). LpoA has an estimated total width of ∼30 Å and length of ∼145 Å. These dimensions should enable the protein to reach PBP1A through the periplasm and cross the ∼60-Å-thick PG layer, which has ∼40- to 60-Å-wide pores (Demchick and Koch, 1996). TP, transpeptidase domain; GT, glycosyltransferase domain; IM, inner membrane; OM, outer membrane.

The NMR analysis of full-length LpoA also indicated that LpoA^N^ is not capable of moving independently of LpoA^C^. Instead, both domains must be connected rather rigidly, consistent with the absence of any flexible stretch in this region. NMR, AUC, and SAXS measurements support an elongated molecular shape of LpoA with a length of ∼145 Å. With this length, LpoA would be capable of spanning from the outer membrane through the periplasm and PG layer to reach a bifunctional PG synthase, such as PBP1A, whose TPase domain is up to ∼100 Å away from the cytoplasmic membrane (Sung et al., 2009; Figure 4). The PG layer is elastic and has pores with a diameter of ∼40 Å in the relaxed state, allowing the diffusion of globular proteins of up to 25 kDa (Demchick and Koch, 1996, Vollmer and Höltje, 2004). The pores in stretched PG may have a diameter of ∼60 Å and allow the diffusion of proteins of up to ∼100 kDa (Demchick and Koch, 1996, Vázquez-Laslop et al., 2001). Hence, LpoA with a width of ∼30 Å should be able to penetrate the pores present in PG. Therefore, our data on the molecular dimensions and shape of LpoA are consistent with the observed crosslinking of LpoA with PBP1A in intact cells (Typas et al., 2010).

The synthesis of new PG and its incorporation into the existing cell wall in growing and dividing bacteria is a well-regulated process, ensuring that the growth of all cell envelope layers is coordinated with cell growth. We are only beginning to understand the complexity of these processes and still lack insights into crucial molecular details of PG growth. PG synthases engage in multiple interactions with other PG enzymes, and the cytoskeletal proteins FtsZ and MreB with associated proteins are essential to guide PG growth (Typas et al., 2012). Presumably, these proteins form large multiprotein complexes for PG synthesis during cell elongation and division, called the elongasome and divisome, respectively (Szwedziak and Löwe, 2013). Bifunctional PG synthases with both glycosyltransferase and transpeptidase activities are part of these complexes and play a major role in PG growth, but only few structures of these important enzymes are known (Han et al., 2011, Lovering et al., 2007, Sung et al., 2009). Hence, we need more structural data on PG enzymes and their interacting proteins. In this report, we have presented data on LpoA that form the structural basis for understanding the activation of PG synthases by outer-membrane proteins.

## Experimental Procedures

### Protein Purification

BL21(DE3) strains harboring pET28LpoA or pET28LpoA^N^ (Typas et al., 2010) were used to produce soluble full-length LpoA or LpoA^N^ (lipid anchor replaced by an oligohistidine tag) with different isotopic labeling schemes (details in Supplemental Experimental Procedures).

### NMR Spectroscopy

NMR data were collected on a 2.5 mM [^13^C, ^15^N]-LpoA^N^ sample in 100 mM sodium acetate buffer, pH 4.5, containing 10% D_2_O at 323 K. Backbone and side-chain resonances were assigned as previously described (Jean et al., 2014). For the assignment of backbone resonances of flexible regions of full-length LpoA, a 3D HNCACB and a 3D BEST-TROSY-(H)N(COCA)NH (Solyom et al., 2013) were collected at 293 K on a 0.75 mM [^13^C, ^15^N]-LpoA sample in 50 mM HEPES, 100 mM NaCl, pH 6.5, containing 10% D_2_O (buffer I), on Bruker spectrometers operating at 700 and 950 MHz ^1^H NMR frequency, equipped with triple ^1^H, ^15^N, ^13^C-resonance cryoprobes. 2D ^1^H-^15^N-BEST-TROSY and 2D-methyl-HMQC/HSQC (Tugarinov et al., 2003) spectra were recorded on the 950-MHz Bruker spectrometer on a 72 μM U-[^2^H, ^12^C, ^15^N], Val-[^13^C^1^H_3_]^*pro-S*^, Ala-[^13^C^1^H_3_]-LpoA, or 297 μM U-[^2^H, ^12^C, ^15^N]-LpoA sample in buffer I at 293 K. Details regarding the data analysis are reported in Supplemental Experimental Procedures.

### Extraction of Structural Restraints and Structure Calculation

Distance restraints from 3D ^15^N-NOESY-HSQC, and 3D aliphatic, aromatic ^13^C-NOESY-HSQC experiments were obtained by using UNIO’10 version 2.0.2 (Guerry and Herrmann, 2012). Additional distance constraints were extracted from peak volumes from a methyl-^13^C-NOESY-HSQC experiment assigned manually. TALOS+ was used to determine phi/psi dihedral angle restraints from chemical shifts (Shen et al., 2009). The structure of LpoA^N^ was calculated with Aria 2.3.1 (Rieping et al., 2007) and 100 structures were calculated in each iteration, except in the last cycle when 700 structures were calculated. The 20 lowest-energy structures underwent explicit water refinement. The structures were visualized graphically and all figures were made with the Pymol Molecular Graphics System, version 1.5.0.4 (Schrödinger, LLC).

### AUC

Sedimentation velocity experiments were performed on an analytical ultracentrifuge XLI (Beckman Coulter) operating at 293 K with a rotor speed of 42,000 rpm. Three ^15^N-labeled LpoA samples (6, 2, and 0.2 mg/ml) in 50 mM HEPES, 100 mM NaCl, pH 6.5, were loaded on double-sector centerpieces in the Anti-50 rotor with optical path lengths of 1.5, 3 and 12 mm, respectively (Nanolytics). The data were acquired at 280 nm using interference optics and analyzed with SEDFIT 14.1 (Schuck, 2000).

### SAXS

SAXS data were collected on beamline BM29 at the European Synchrotron Radiation Facility (Grenoble, France). Scattering data were collected on three ^15^N-LpoA samples with concentrations of 5, 2, and 1 mg/ml, in the same buffer conditions as in the AUC experiments. Each sample was positioned at 2.87 m from a Pilatus detector and ten frames of 1 s were recorded at a wavelength of 0.99 Å. After normalization to the intensity of the transmitted beam, frames were merged for each sample. Subtraction of the buffer’s contribution to the scattering and further processing steps were performed with PRIMUS from the ATSAS 2.5.1 program package (Svergun, 1992). The radius of gyration, R_g_, forward scattering intensity, I(0), maximum particle dimension, D_max_, and distance distribution function, P(r) were evaluated with GNOM. The R_g_ was also estimated using the Guinier approximation in the Autorg software (Petoukhov et al., 2007). The NMR structure of LpoA^N^ and a model structure of *E. coli* LpoA^C^, predicted by PHYRE (Kelley and Sternberg, 2009) from its homolog in *H. influenzae*, were used to calculate the R_g_ and P(r). Globular, L-shaped, and elongated models of full-length LpoA were created using different dihedral angles in the linker. Scattering curves were then simulated on these models by CRYSOL. Their respective distance distribution function and the parameters R_g_ and D_max_ were calculated by GNOM.

## Accession Numbers

The coordinates of 20 structures and chemical shifts of LpoA^N^ have been deposited in the Protein Data Bank and the BioMagResBank under accession codes 2MHK and 18853, respectively.

## Supplemental Information

Document S1. Supplemental Experimental Procedures and Figures S1–S3

Document S2. Article plus Supplemental Information

## References

[bib1] Allan R.K., Ratajczak T. (2011). Versatile TPR domains accommodate different modes of target protein recognition and function. Cell Stress Chaperones.

[bib2] Banzhaf M., van den Berg van Saparoea B., Terrak M., Fraipont C., Egan A., Philippe J., Zapun A., Breukink E., Nguyen-Distèche M., den Blaauwen T., Vollmer W. (2012). Cooperativity of peptidoglycan synthases active in bacterial cell elongation. Mol. Microbiol..

[bib3] Bertsche U., Breukink E., Kast T., Vollmer W. (2005). *In vitro* murein peptidoglycan synthesis by dimers of the bifunctional transglycosylase-transpeptidase PBP1B from *Escherichia coli*. J. Biol. Chem..

[bib4] Biegert A., Mayer C., Remmert M., Söding J., Lupas A.N. (2006). The MPI Bioinformatics Toolkit for protein sequence analysis. Nucleic Acids Res..

[bib5] Born P., Breukink E., Vollmer W. (2006). In vitro synthesis of cross-linked murein and its attachment to sacculi by PBP1A from *Escherichia coli*. J. Biol. Chem..

[bib6] Demchick P., Koch A.L. (1996). The permeability of the wall fabric of *Escherichia coli* and *Bacillus subtilis*. J. Bacteriol..

[bib7] Guerry P., Herrmann T. (2012). Comprehensive automation for NMR structure determination of proteins. Methods Mol. Biol..

[bib8] Han S., Caspers N., Zaniewski R.P., Lacey B.M., Tomaras A.P., Feng X., Geoghegan K.F., Shanmugasundaram V. (2011). Distinctive attributes of β-lactam target proteins in Acinetobacter baumannii relevant to development of new antibiotics. J. Am. Chem. Soc..

[bib9] Holm L., Rosenström P. (2010). Dali server: conservation mapping in 3D. Nucleic Acids Res..

[bib10] Jean N.L., Bougault C., Derouaux A., Callens G., Vollmer W., Simorre J.P. (2014). Backbone and side-chain ^1^H, ^13^C, and ^15^N NMR assignments of the N-terminal domain of *Escherichia coli* LpoA. Biomol. NMR Assign..

[bib11] Kelley L.A., Sternberg M.J. (2009). Protein structure prediction on the Web: a case study using the Phyre server. Nat. Protoc..

[bib12] Kim K.H., Aulakh S., Paetzel M. (2011). Crystal structure of β-barrel assembly machinery BamCD protein complex. J. Biol. Chem..

[bib13] Lovering A.L., de Castro L.H., Lim D., Strynadka N.C. (2007). Structural insight into the transglycosylation step of bacterial cell-wall biosynthesis. Science.

[bib14] Paradis-Bleau C., Markovski M., Uehara T., Lupoli T.J., Walker S., Kahne D.E., Bernhardt T.G. (2010). Lipoprotein cofactors located in the outer membrane activate bacterial cell wall polymerases. Cell.

[bib15] Petoukhov M.V., Konarev P.V., Kikhney A.G., Svergun D.I. (2007). ATSAS 2.1—towards automated and web-supported small-angle scattering data analysis. J. Appl. Cryst..

[bib16] Rieping W., Habeck M., Bardiaux B., Bernard A., Malliavin T.E., Nilges M. (2007). ARIA2: automated NOE assignment and data integration in NMR structure calculation. Bioinformatics.

[bib17] Sandoval C.M., Baker S.L., Jansen K., Metzner S.I., Sousa M.C. (2011). Crystal structure of BamD: an essential component of the β-Barrel assembly machinery of Gram-negative bacteria. J. Mol. Biol..

[bib18] Schuck P. (2000). Size-distribution analysis of macromolecules by sedimentation velocity ultracentrifugation and lamm equation modeling. Biophys. J..

[bib19] Shen Y., Delaglio F., Cornilescu G., Bax A. (2009). TALOS+: a hybrid method for predicting protein backbone torsion angles from NMR chemical shifts. J. Biomol. NMR.

[bib20] Solyom Z., Schwarten M., Geist L., Konrat R., Willbold D., Brutscher B. (2013). BEST-TROSY experiments for time-efficient sequential resonance assignment of large disordered proteins. J. Biomol. NMR.

[bib21] Sung M.T., Lai Y.T., Huang C.Y., Chou L.Y., Shih H.W., Cheng W.C., Wong C.H., Ma C. (2009). Crystal structure of the membrane-bound bifunctional transglycosylase PBP1b from *Escherichia coli*. Proc. Natl. Acad. Sci. USA.

[bib22] Svergun D.I. (1992). Determination of the regularization parameter in indirect-transform methods using perceptual criteria. J. Appl. Cryst..

[bib23] Szwedziak P., Löwe J. (2013). Do the divisome and elongasome share a common evolutionary past?. Curr. Opin. Microbiol..

[bib24] Tugarinov V., Hwang P.M., Ollerenshaw J.E., Kay L.E. (2003). Cross-correlated relaxation enhanced 1H-13C NMR spectroscopy of methyl groups in very high molecular weight proteins and protein complexes. J. Am. Chem. Soc..

[bib25] Typas A., Banzhaf M., van den Berg van Saparoea B., Verheul J., Biboy J., Nichols R.J., Zietek M., Beilharz K., Kannenberg K., von Rechenberg M. (2010). Regulation of peptidoglycan synthesis by outer-membrane proteins. Cell.

[bib26] Typas A., Banzhaf M., Gross C.A., Vollmer W. (2012). From the regulation of peptidoglycan synthesis to bacterial growth and morphology. Nat. Rev. Microbiol..

[bib27] Vázquez-Laslop N., Lee H., Hu R., Neyfakh A.A. (2001). Molecular sieve mechanism of selective release of cytoplasmic proteins by osmotically shocked *Escherichia coli*. J. Bacteriol..

[bib28] Vijayalakshmi J., Akerley B.J., Saper M.A. (2008). Structure of YraM, a protein essential for growth of *Haemophilus influenzae*. Proteins.

[bib29] Vollmer W., Höltje J.-V. (2004). The architecture of the murein (peptidoglycan) in Gram-negative bacteria: vertical scaffold or horizontal layer(s)?. J. Bacteriol..

[bib30] Vollmer W., Blanot D., de Pedro M.A. (2008). Peptidoglycan structure and architecture. FEMS Microbiol. Rev..

[bib31] Zeytuni N., Zarivach R. (2012). Structural and functional discussion of the tetra-trico-peptide repeat, a protein interaction module. Structure.

