# Supplementary material for: Elongated Structure of the Outer-Membrane Activator of Peptidoglycan Synthesis LpoA: Implications for PBP1A Stimulation
Source: Structure. 2014 Jul 8;22(7):1047–54. doi: 10.1016/j.str.2014.04.017 (PMC4111904; doi:10.1016/j.str.2014.04.017)
Supplement: Document S1. Supplemental Experimental Procedures and Figures S1–S3 [file mmc1.pdf]

Structure, Volume *22*

Supplemental Information

Elongated Structure of the Outer-Membrane Activator of Peptidoglycan Synthesis LpoA: Implications for PBP1A Stimulation

Nicolas L. Jean, Catherine M. Bougault, Adam Lodge, Adeline Derouaux, Gilles Callens, Alexander J.F. Egan, Isabel Ayala, Richard J. Lewis, Waldemar Vollmer, and Jean-Pierre Simorre

SUPPLEMENTAL INFORMATION

The elongated structure of the outer-membrane activator of peptidoglycan synthesis LpoA: implications in PBP1A-stimulation

Nicolas L. Jean, Catherine M. Bougault, Adam Lodge, Adeline Derouaux, Gilles Callens, Alexander J. F. Egan, Isabel Ayala, Richard J. Lewis, Waldemar Vollmer,* Jean-Pierre Simorre*

Inventory of Supplemental Information

Supplemental Experimental Procedures **2**

Expression and purification of LpoA versions. 2

Secondary structure propensities in the flexible regions 1 and 2 of full-length LpoA 4

NMR data processing and analysis. 5

Supplemental References **5**

Supplemental Figures

Figure S1, related to Figure 1 **7**

Characterization of $LpoA^N$ and full-length LpoA in different conditions.

Figure S2, related to Figure 2 **9**

Characteristic spectroscopic signatures of $LpoA^N$ and full-length LpoA.

Figure S3, related to Figure 3 **12**

Guignier and Kratky plots calculated from SAXS data measured on a 5 mg/mL LpoA sample.

Supplemental Experimental Procedures

Expression and purification of LpoA versions.

Unlabeled LpoA and $LpoA^N$. BL21(DE3) strains harboring plasmids pET28LpoA or $pET28LpoA^N$ were used to purify soluble full-length LpoA or $LpoA^N$ (lipid anchor replaced by an oligohistidine tag). Three liters of LB medium (10 g/L tryptone, 5 g/L yeast extract, 10 g/L NaCl) containing 50 µg/mL kanamycin were inoculated 1 in 50 with a corresponding overnight culture and incubated at 37°C until the OD_{578} reached 0.4 – 0.6. Overexpression of recombinant *lpoA* genes was induced by the addition of 1 mM IPTG and further incubation for 3 h at 30°C. Cells were harvested by centrifugation (10,000 × g, 15 min, 4°C) and the pellet was resuspended in 40 mL of buffer I (25 mM Tris/HCl, 10 mM $MgCl_2$, 500 mM NaCl, 20 mM imidazole, 10% glycerol, pH 7.5). DNase, protease inhibitor cocktail (Sigma) (1 to 1000 dilution) and 100 µM phenylmethylsulfonylfluoride (PMSF) was added before cells were disrupted by sonication using a Branson digital sonicator. The lysate was centrifuged (130,000 × g, 60 min, 4°C) and the supernatant was applied at a 1 mL/min flow rate to a 5 mL HisTrap HP column (GE Healthcare) attached to an ÄKTA PrimePlus (GE Healthcare) FPLC. The column was washed with 4 volumes of buffer I before step-wise elution of bound proteins with buffer II (25 mM Tris/HCl, 10 mM $MgCl_2$, 500 mM NaCl, 400 mM imidazole, 10% glycerol, pH 7.5). If a second purification step was required, proteins were dialyzed against IEX buffer A (20 mM Tris/HCl, pH 8.0) and applied to a 5 mL HiTrap Q HP column (GE healthcare) at a flow rate of 0.5 mL/min using an ÄKTA PrimePlus FPLC system. The column was washed with 85% IEX buffer A and 15% IEX buffer B (20 mM Tris/HCl, 500 mM NaCl, pH 8.0) for 10 column volumes at 2 mL/min before a linear gradient from 15% to 100% B over 150 mL was applied at 2 mL/min. The eluted LpoA protein was pooled and concentrated to 4 – 5 mL for application to a Superdex200 HiLoad 16/600 column at 1 mL/min for size exclusion chromatography in a buffer containing 25 mM Tris/HCl, 10 mM $MgCl_2$, 500 mM NaCl, 10% glycerol at pH 7.5. In the case of $LpoA^N$ the second purification step (ion exchange chromatography) was omitted.

U-^{15}N-LpoA, U-[^{13}C, ^{15}N]-LpoA and U-[^{13}C, ^{15}N]-LpoA^{N}. For the production of ^{15}N- or ^{13}C,^{15}N-isotopically labeled versions, 2 L M9 growth medium (5.29 g/L Na_2HPO_4, 3 g/L KH_2PO_4, 0.5 g/L NaCl, 1 g/L NH_4Cl, 2 mM thiamine, 1 mM $MgSO_4$, 0.1 mM $CaCl_2$, 0.3% glucose, pH 6.8 – 7.2) containing 50 µg/mL kanamycin were used. For single labeled protein [^{15}N]-NH_4Cl was used, for double labeled protein both [^{15}N]-NH_4Cl and [^{13}C]-glucose were used (Cambridge isotope laboratories Inc, USA). Cells were grown overnight in 100 mL M9 medium per liter of final culture, harvested by centrifugation (3,000 × g, 20 min, RT) and resuspended in 2 × 1 mL of fresh M9 medium which was used to inoculate the remaining 900 mL M9 medium containing the desired isotopically labelled compound(s). Purification proceeded by affinity and gel filtration chromatography as described above.

Perdeuterated LpoA samples. The U-[^{2}H, ^{15}N, ^{12}C], Val-[2,3-^{2}H$_2$; 1,2,3-^{12}C$_3$; [^{12}C^{2}H$_3$]$^{pro\text{-}R}$/[^{13}C^{1}H$_3$]$^{pro\text{-}S}$], Ala-[2-^{12}C^{2}H; 3-^{13}C^{1}H$_3$]-LpoA sample (labeled in short U-[^{2}H, ^{15}N, ^{12}C], Val-[^{13}C^{1}H$_3$]$^{pro\text{-}S}$], Ala-[^{13}C^{1}H$_3$]-LpoA) was prepared according to our published protocols (Ayala et al., 2009; Mas et al., 2013). *E. coli* BL21(DE3) carrying the LpoA plasmid were progressively adapted in three stages over 24 h to an enriched M9/D_2O medium containing 5.3 g/L Na_2HPO_4, 3 g/L KH_2PO_4, 0.5 g/L NaCl, 1 g/L $^{15}NH_4Cl$, 1 mM thiamine, 1 mM $MgSO_4$, 0.1 mM $CaCl_2$, 50 µM $ZnSO_4$, 100 µM $FeCl_3$, 30 mg/L kanamycin, a vitamin cocktail, and 2 g/L D-glucose-d$_7$ ($^{12}C_6{}^{1}H_5{}^{2}H_7O_6$, Isotec) at pH 6.8 – 7.2. Two 500 mL cultures in enriched M9/D_2O medium were inoculated and grown at 37°C. When the OD_{600} reached 0.6 to 0.8, solutions of [2-^{12}C^{2}H; 3-^{13}C^{1}H$_3$]-L-alanine (NMR-Bio), *pro-S* acetolactate-^{13}C (2-hydroxy-2-[^{13}C]methyl-3-oxo-4,4,4-tri-[^{2}H]butanoate, NMR-Bio) and L-leucine-d$_{10}$ (Sigma-Aldrich) in D_2O were added to final concentrations of 500 mg/L, 240 mg/L and 30 mg/L, respectively. After one hour, protein expression was induced by the addition of 1 mM IPTG and the culture was incubated at 20°C overnight. Cells were harvested by centrifugation (5,000 × g, 10 min, 4°C) and the pellet was resuspended in 40 mL of buffer I (25 mM Tris/HCl, 100 mM NaCl, pH 7.5). Purification proceeded through affinity and gel filtration chromatography steps as described above. To prevent LpoA sample degradation, purification steps were led at 4°C in

presence of Complete™ protease inhibitor cocktail (Roche).

The U-[^{2}H, ^{15}N, ^{12}C]-LpoA sample was prepared according to the protocol described by Rasia et al. in 2009. *E. coli* BL21(DE3) bacterial cells harboring the LpoA plasmid were grown in M9/H_2O medium containing 10 g/L $Na_2HPO_4.7H_2O$, 3 g/L KH_2PO_4, 0.5 g/L NaCl, 1 g/L $^{15}NH_4Cl$, 1 mM thiamine, 1 mM $MgSO_4$, 0.1 mM $CaCl_2$, 50 μM $ZnSO_4$, 100 μM $FeCl_3$, 30 mg/L kanamycin, a vitamin cocktail, and 2 g/L D-glucose-d$_7$ ($^{12}C_6{}^1H_5{}^2H_7O_6$, Isotec) at 37°C. When the OD_{600} reached 0.5 to 0.6, the medium was supplemented with 3 g/L -[^{2}H, ^{15}N, ^{12}C]-ISOGRO™ powder (Isotec). After one hour, 1 mM IPTG was added and the culture was incubated at 20°C overnight. The purification protocol was identical to the one described just above. Due to the presence of H_2O instead of D_2O in the culture medium, all the water exchangeable nuclei remained protonated, in contrast to the previous sample.

Both perdeuterated protein samples were characterized by gel electrophoresis and the HPLC profile from the gel filtration chromatography and showed identical properties as the U-[^{1}H, ^{13}C, ^{15}N] sample.

Secondary structure propensities in the flexible regions 1 and 2 of full-length LpoA.

The ^{1}H, ^{15}N and ^{13}C chemical shifts from assigned residues in the flexible regions 1 (N285 – P351) and 2 (S493 – N531) of full-length LpoA have been analyzed to determine secondary structure propensity. Chemical shift differences with respect to random coil values corrected with sequence-dependent effects (Schwarzinger et al., 2001) failed to identify secondary structure elements. The SSP software (Marsh et al., 2006) computed 10.5% and 1.4% β-structure and 0.1% and 1.0% α-helical propensity for regions 1 and 2, respectively. MICS (Shen and Bax, 2012) and TALOS+ (Shen et al., 2009) showed marked dynamics for regions 1 and 2 with S^2 values of 0.449 ± 0.091 and 0.458 ± 0.105. These predictions are consistent with the either positive and below 0.2 or negative {^{1}H}-^{15}N-NOE values measured for the sequence specifically assigned residues. The latter programs predicted a higher uniformly distributed β-structure propensity in regions 1 and 2

(28% and 24%, respectively). Delta2D (Camilloni et al., 2012), developed specifically for the analysis of disordered proteins, gave 6.1% and 7.5% β-structure and 87.5% and 83.8% coil propensity for regions 1 and 2, respectively. Consequently, regions 1 and 2 appear disordered or dynamic, at least for the assigned residues, which include a large portion of region 1 and the loop region of region 2. The secondary structure in these regions cannot be determined conclusively from the different programs used because of the discrepancies of their outputs, which may in part be correlated to the incompleteness of the specific resonance assignments for these regions.

NMR data processing and analysis.

NMR data were processed with NMRPipe (Delaglio et al., 1995) and analyzed using the CcpNmr Analysis 2.2 software (Vranken et al., 2005). To estimate correlation times of the N-terminal and C-terminal domain of full-length LpoA, the intensity of ^{1}H-^{13}C correlations of methyl groups for alanine residues in the HMQC (I_{HMQC}) and HSQC (I_{HSQC}) spectra were determined, after fitting of the individual lineshapes with a Gaussian function within the CcpNmr software. Intensity ratios I_{HMQC}/I_{HSQC} were calculated for each individual methyl group. Similar ratios (average value 1.3) were obtained for resolved alanine correlations arising from the N-terminal and C-terminal domains, suggesting a similar correlation time for the two domains. These ratios were drastically different and showed a systematic enhancement with respect to ratios calculated for residues arising from the flexible regions 1 and 2 in the C-terminal domain.

Supplemental References

Ashkenazy, H., Erez, E., Martz, E., Pupko, T., and Ben-Tal, N. (2010). ConSurf 2010: calculating evolutionary conservation in sequence and structure of proteins and nucleic acids. Nucleic Acids Res. *38*, W529-W533.

Ayala, I., Sounier, R., Usé, N., Gans, P., and Boisbouvier, J. (2009). An efficient protocol for the complete incorporation of methyl-protonated alanine in perdeuterated protein. J Biomol NMR, *43*, 111-119.

Camilloni, C., De Simone, A., Vranken, W., and Vendruscolo,, M. (2012). Determination of secondary structure populations in disordered states of proteins using NMR chemical shifts. Biochemistry *51*, 2224-2231.

Delaglio, F., Grzesiek, S., Vuister, G.W., Zhu, G., Pfeifer, J., and Bax, A. (1995). NMRPipe: a multidimensional spectral processing system based on UNIX pipes. J Biomol NMR *6*, 277-293.

Marsh, J.A., Singh, V.K., Jia, Z. and Forman-Kay, J.D. (2006). Sensitivity of secondary structure propensities to sequence differences between α- and γ-synuclein: Implications for fibrillation. Protein Science *15*, 2795–2804.

Mas, G., Crublet, E., Hamelin, O., Gans, P., and Boisbouvier, J. (2013). Specific labeling and assignment strategies of valine methyl groups for NMR studies of high molecular weight proteins. J Biomol NMR, *57*, 251-262.

Rasia, R.M., Noirclerc-Savoye, M., Bologna, N.G., Gallet, B., Plevin, M.J., Blanchard, L., Palatnik, J.F., Brutscher, B., Vernet, T., and Boisbouvier, J. (2009). Parallel screening and optimization of protein constructs for structural studies. Protein Sci *18*, 434-439.

Shen, Y., Delaglio, F., Cornilescu, G., and Bax, A. (2009). TALOS+: a hybrid method for predicting protein backbone torsion angles from NMR chemical shifts. J Biomol NMR *44*, 213-223.

Shen, Y., and Bax, A. (2012). Identification of helix capping and beta-turn motifs from NMR chemical shifts. J. Biomol. NMR *52*, 211-232.

Schwarzinger, S., Kroon, G.J.A., Foss, T.R., Chung J., Wright, P.E., and Dyson, H.J. (2001). Sequence-dependent correction of random coil NMR chemical shifts. J Am Chem Soc *123*, 2970-2978.

Vranken, W.F., Vriend, G., and Vuister, G.W. (2012). CING: an integrated residue-based structure validation program suite. J Biomol NMR *54*, 267-283.

Figure S1

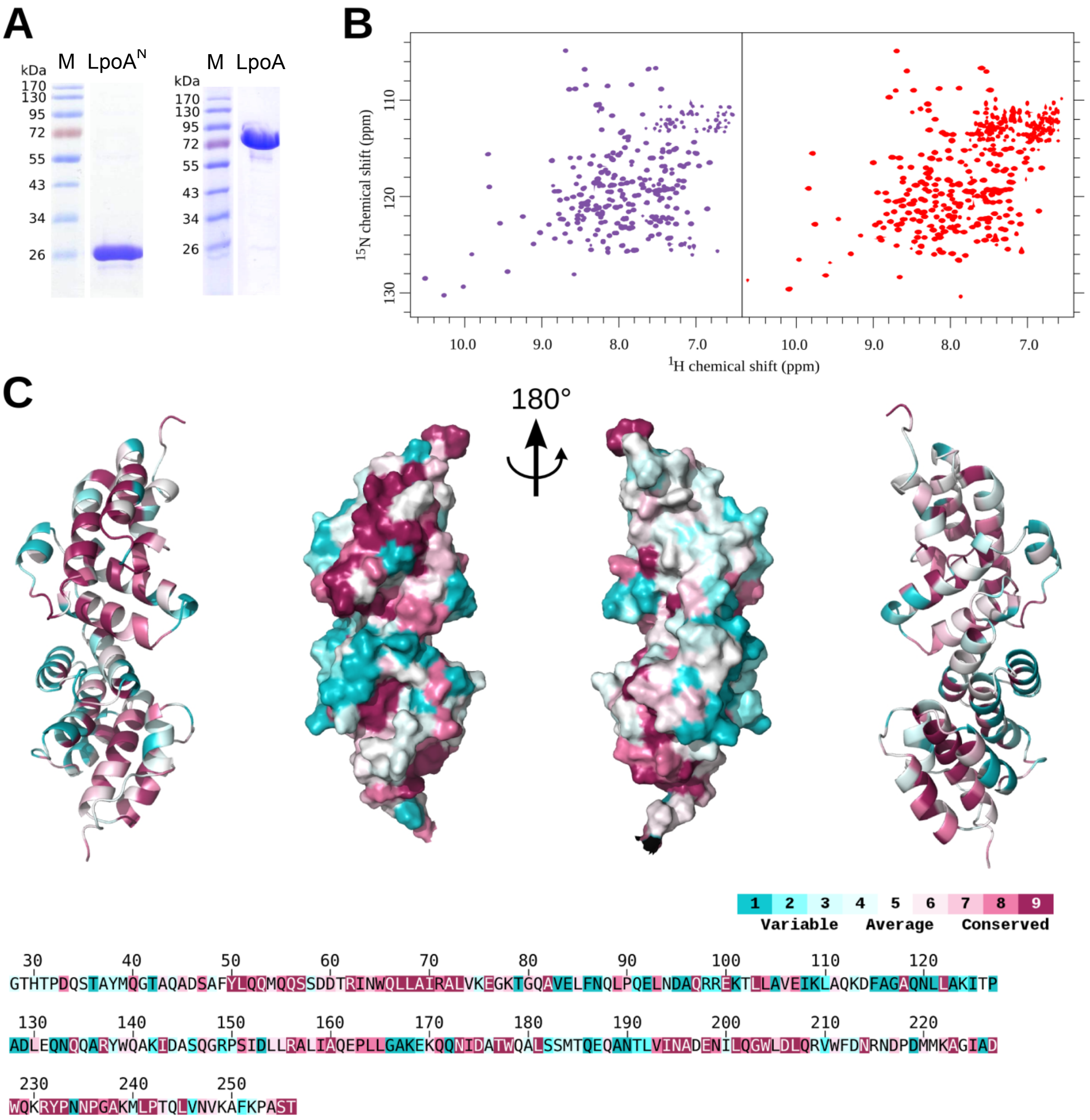

Figure S1, related to Figure 1: Characterization of LpoA^N and full-length LpoA in different conditions. (A) SDS-PAGE analysis of purified LpoA^N and LpoA. The purified proteins were separated by SDS-PAGE before the gels were stained with Coomassie Blue. The molecular weight of the protein markers shown in lanes M is listed. (B) The stability of LpoA^N in different temperature and pH conditions was evaluated by NMR. ^{1}H-^{15}N-BEST-TROSY spectrum recorded at 50°C and pH 4.5 in 150 mM sodium acetate buffer is shown in the left panel (purple). The equivalent spectrum recorded at 25°C and pH 7.0 after dialysis of the same LpoA^N sample in 20 mM HEPES buffer is shown in the right panel (red). Additional conditions (10 mM TRIS buffer at pH 7.5 and 25°C, 20 mM HEPES buffer containing 100 mM NaCl at pH 7.0 and 25°C, 100 mM

MOPS at pH 6.9 and 25°C, 100 mM MES buffer at pH 6.3 and 35°C, 200 mM sodium acetate buffer at pH 4.5 and 35°C) were investigated (data not shown). LpoA^N was stable from 5 to 50°C and on the [4.5 – 7.5] pH range, as emphasized from the characteristic NMR peak pattern. The signal-to-noise ratio in the NMR spectra is increased at elevated temperature and acidic pH. (C) Conserved residues in LpoA^N identified by the Consurf server (Ashkenazy et al., 2010). Multiple sequence alignment was built for LpoA^N (residues 28 to 256) from 63 unique protein sequences using MAFFT and sequences from homologues were obtained from the UNIREF90 database and the CSI-BLAST algorithm. Conservation scores obtained from this alignment were used to color residues on a cartoon and a surface representation of the LpoA^N structure. Two orientations rotated by 180° are shown for the two types of representations. On the bottom part of this panel, the conservation scores are reported on the primary sequence of the LpoA^N protein.

Figure S2

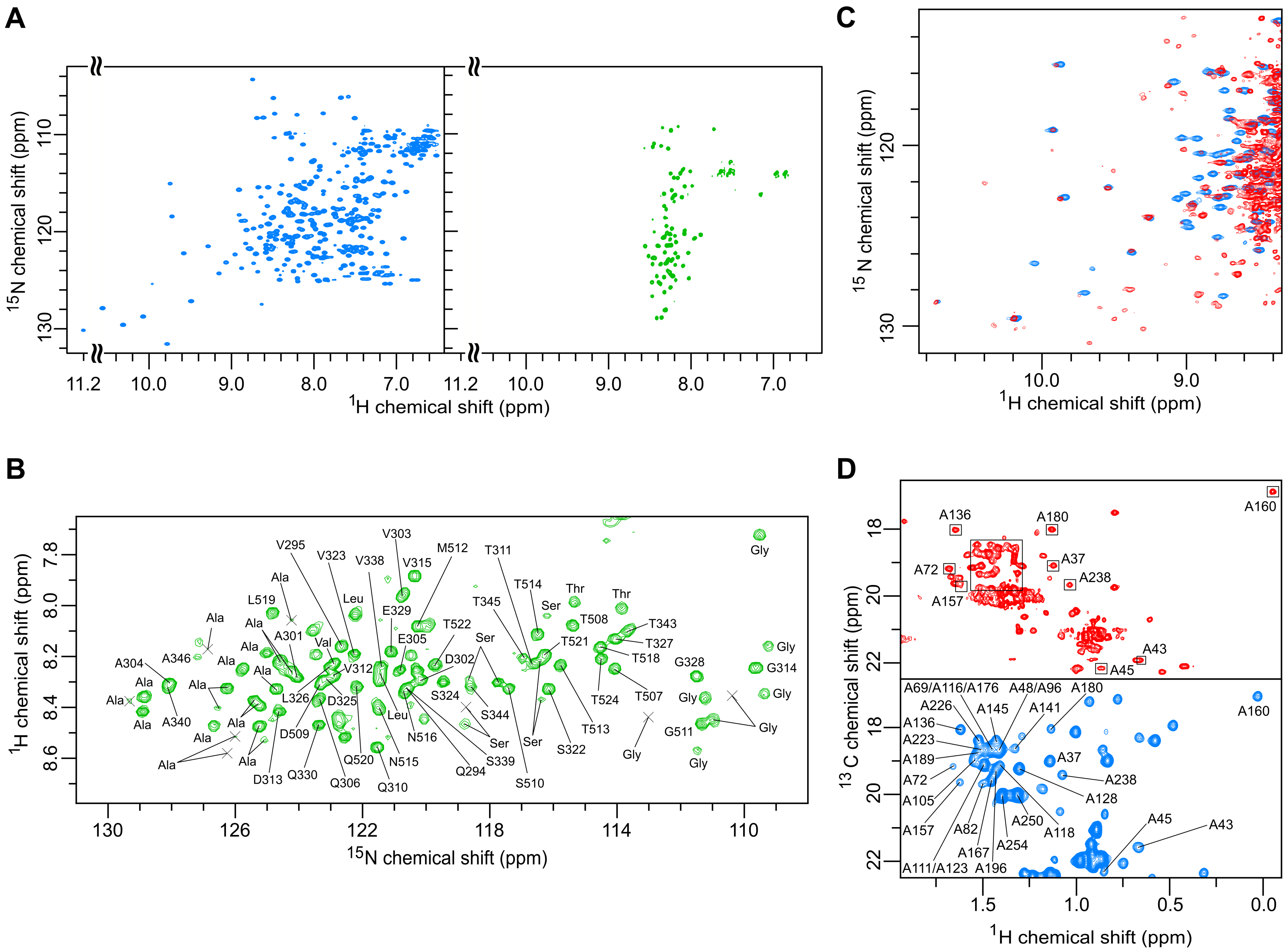

A
B
C
D
^{15}N chemical shift (ppm)
^{1}H chemical shift (ppm)
^{13}C chemical shift (ppm)
V295
V323
V338
V303
V315
M512
T311
T514
Thr
Ser
T345
T508
T343
T327
T518
T521
T522
E329
E305
D302
Ser
L519
A301
Val
Leu
V312
L326
D325
S324
S344
Gly
G314
G328
G511
A304
A346
A340
Ala
D509
D313
Q330
Q306
Q520
N515
Q310
Q294
S339
S510
S322
T513
T524
T507
N516
A136
A180
A160
A37
A72
A238
A157
A43
A45
A69/A116/A176
A48/A96
A226
A145
A141
A223
A189
A105
A128
A82
A250
A118
A167
A254
A111/A123
A196

Figure S2, related to Figure 2. Characteristic spectroscopic signatures of $LpoA^N$ and full-length LpoA. (A) The ^1H-^{15}N-HSQC spectrum (left, blue) on $LpoA^N$ was recorded at 50°C and pH 4.5 in a 100 mM sodium acetate buffer. The ^1H-^{15}N-BEST-TROSY spectrum (right, green) on ^{13}C,^{15}N-LpoA was recorded at 20°C and pH 6.5 in a 50 mM HEPES, 100 mM NaCl buffer. In this spectrum, only the amide resonances of residues located in flexible portions (mainly additional regions 1 and 2) were detected. (B) Portion of the LpoA spectrum shown in the previous right panel after rotation of the axes. Assignment of the resonances of disordered residues in LpoA was pursued with the collection of 3D HNCACB and BEST-TROSY-(H)N(COCA)NH. Residues sequentially assigned are indicated with the amino acid numbering of the wild-type LpoA protein. For the residues not sequentially assigned, the residue type obtained from the Cα and Cβ chemical shifts (for 36 resonances among which 19 Ala, which fits reasonably well with the 14 unassigned Ala in region 1 and the 2 Ala in region 2) is indicated by the name of the corresponding amino acid. Peaks observable only at lower threshold are marked with a cross. When the protein sample was stored for a few weeks at 4°C, the full-length ^{13}C,^{15}N-LpoA sample showed degradation of the C-terminal domain (as confirmed by gel electrophoresis and mass spectrometry) and reappearance of the signature of $LpoA^N$. At 20°C, the sample showed significant degradation within 48 h. (C) The left panel shows an overlay of the resolved portion of the 2D ^1H-^{15}N-BEST-TROSY (red) and ^1H-^{15}N-HSQC (blue) amide region collected on 72 μM U-[^2H, ^{12}C, ^{15}N], Val-[$^{13}C^1H_3$]$^{pro\text{-}S}$, Ala-[$^{13}C^1H_3$]-LpoA and 200 μM ^{13}C,^{15}N- $LpoA^N$ samples, respectively, in 50 mM HEPES, 100 mM NaCl containing 10% D_2O at pH 6.5 and 293 K. This spectrum readily allows the identification of the N-terminal domain resonances in the full-length LpoA sample. ^1H-^{15}N correlations exclusively present in the full-length LpoA sample arise from the folded C-terminal domain for the main part, while missing correlations may arise from the partial reprotonation of amide positions as the sample was produced in a D_2O medium. (D) The top spectrum (red) shows a portion of the ^1H-^{13}C-methyl selective HMQC collected on the U-[^2H, ^{12}C, ^{15}N], Val-[$^{13}C^1H_3$]$^{pro\text{-}S}$, Ala-[$^{13}C^1H_3$]-LpoA sample in 50 mM HEPES, 100 mM NaCl containing 10% D_2O at pH 6.5 at 293K. Assignments of 27 out of

the 29 resonances of the N-terminal domain of LpoA could be transferred from the bottom spectrum (blue). This spectrum corresponds to a portion of the ^{1}H-^{13}C-CT-HSQC recorded at 50°C and pH 4.5 on the LpoA^{N} sample in a 100 mM sodium acetate buffer. In the latter spectrum only Ala β resonances are labeled; Ile γ2, Val γ1 and γ2, and Thr γ resonances are kept unlabeled for clarity.

Figure S3

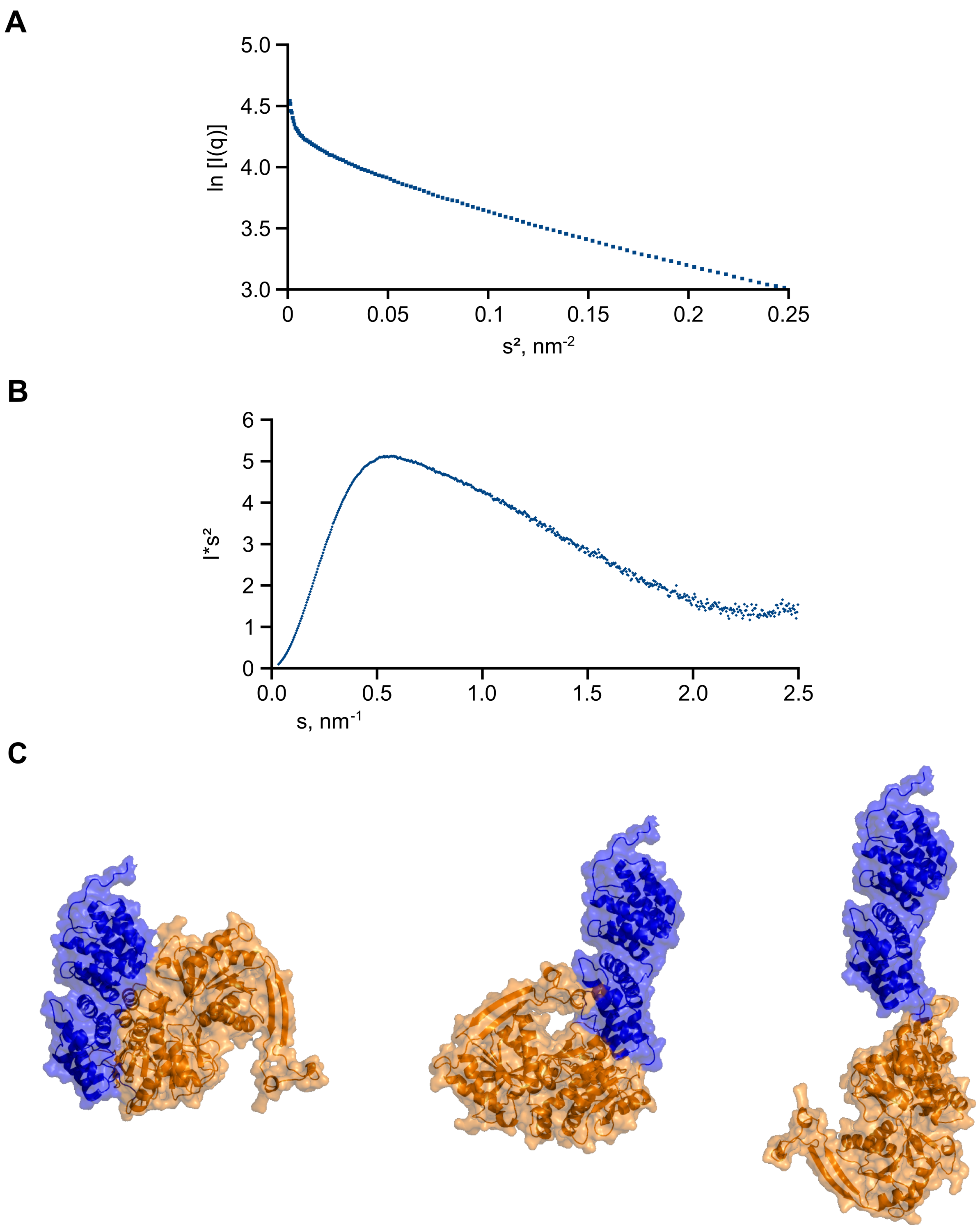

Figure S3, related to Figure 3. Complementary analysis of SAXS data collected on a 5 mg/mL LpoA sample. (A) The Guinier plot calculated from these data is consistent with a 73.4 kDa monomeric protein. (B) The Kratky profile is consistent with a partially unfolded protein, consistent

with AUC and NMR data discussed in the main text. (C) Ribbon representation of the globular (left), L-shaped (middle) and elongated (right) molecular models of LpoA (used to calculate the P(r) curves shown in Figure 3B). These models were built from the *E. coli* $LpoA^N$ NMR structure (blue) and the *E. coli* $LpoA^C$ structure (orange, modeled from the crystal structure of *H. influenzae* $LpoA^C$ (PDB code 3CKM) with PHYRE) by using different dihedral angles in the linker (Figure 2D).
