# Supplementary material for: Elongated Structure of the Outer-Membrane Activator of Peptidoglycan Synthesis LpoA: Implications for PBP1A Stimulation
Source: Structure. 2014 Jul 8;22(7):1047–54. doi: 10.1016/j.str.2014.04.017 (PMC4111904; doi:10.1016/j.str.2014.04.017)
Supplement: Document S2. Article plus Supplemental Information [file mmc2.pdf]

Structure
Short Article

CellPress

Elongated Structure of the Outer-Membrane Activator of Peptidoglycan Synthesis LpoA: Implications for PBP1A Stimulation

Nicolas L. Jean,[1,2,3] Catherine M. Bougault,[1,2,3] Adam Lodge,[4] Adeline Derouaux,[4,6] Gilles Callens,[4,7] Alexander J.F. Egan,[4] Isabel Ayala,[1,2,3] Richard J. Lewis,[5] Waldemar Vollmer,[4,*] and Jean-Pierre Simorre[1,2,3,*]
[1]University Grenoble Alpes, Institut de Biologie Structurale, F-38027 Grenoble, France
[2]CEA, DSV, Institut de Biologie Structurale, F-38027 Grenoble, France
[3]CNRS, Institut de Biologie Structurale, F-38027 Grenoble, France
[4]The Centre for Bacterial Cell Biology, Institute for Cell and Molecular Biosciences, Newcastle University, Richardson Road, Newcastle upon Tyne NE2 4AX, UK
[5]Institute for Cell and Molecular Biosciences, Newcastle University, Framlington Place, Newcastle upon Tyne NE2 4HH, UK
[6]Present address: Centre d'Ingénierie des Protéines, Université de Liège, allée de la Chimie, B6a, Sart Tilman, 4000 Liège, Belgium
[7]Present address: Eurogentec S.A., 5 rue du Bois Saint Jean, 4102 Seraing, Belgium
*Correspondence: w.vollmer@ncl.ac.uk (W.V.), jean-pierre.simorre@ibs.fr (J.-P.S.)
http://dx.doi.org/10.1016/j.str.2014.04.017

SUMMARY

The bacterial cell envelope contains the stress-bearing peptidoglycan layer, which is enlarged during cell growth and division by membrane-anchored synthases guided by cytoskeletal elements. In *Escherichia coli*, the major peptidoglycan synthase PBP1A requires stimulation by the outer-membrane-anchored lipoprotein LpoA. Whereas the C-terminal domain of LpoA interacts with PBP1A to stimulate its peptide crosslinking activity, little is known about the role of the N-terminal domain. Herein we report its NMR structure, which adopts an all-α-helical fold comprising a series of helix-turn-helix tetratricopeptide-repeat (TPR)-like motifs. NMR spectroscopy of full-length LpoA revealed two extended flexible regions in the C-terminal domain and limited, if any, flexibility between the N- and C-terminal domains. Analytical ultracentrifugation and small-angle X-ray scattering results are consistent with LpoA adopting an elongated shape, with dimensions sufficient to span from the outer membrane through the periplasm to interact with the peptidoglycan synthase PBP1A.

INTRODUCTION

Most bacteria surround their cytoplasmic membrane with a peptidoglycan (PG) sacculus, which maintains cell shape and protects the cell from lysis due to turgor. The PG sacculus is an elastic, net-like molecule composed of glycan chains that are connected by short peptides (Vollmer et al., 2008). Gram-negative bacteria, such as *Escherichia coli*, have a thin, mainly single-layered PG and an outer membrane containing lipopolysaccharide.

Growing and dividing cells polymerize the PG precursor lipid II and insert it into the existing PG layer by the combined actions of PG synthases and hydrolases, which presumably form dynamic, membrane-attached multienzyme complexes (Vollmer et al., 2008). *E. coli* has six cytoplasmic-membrane-anchored PG synthases. PBP1A and PBP1B are the major (and semi-redundant) bifunctional glycosyltransferase-transpeptidases, with well characterized in vitro activities (Banzhaf et al., 2012; Bertsche et al., 2005; Born et al., 2006). PBP2 and PBP3 are transpeptidases that are essential for cell elongation and division, respectively.

PG synthesis and hydrolysis are regulated from inside the cell by cytoskeletal elements (Typas et al., 2012). In *E. coli* and presumably in other Gram-negative bacteria, PG synthesis is also regulated from outside the sacculus. PBP1A and PBP1B both require cognate outer-membrane-anchored lipoproteins (LpoA and LpoB, respectively) for function (Paradis-Bleau et al., 2010; Typas et al., 2010). LpoA and LpoB are unrelated in amino acid sequence, but both attach to the inner leaflet of the outer membrane by an N-terminal lipid modification. The C-terminal domain of LpoA interacts with PBP1A to stimulate its transpeptidase activity by an as yet unknown mechanism, whereas LpoB interacts with the noncatalytic UB2H domain of PBP1B to stimulate its glycosyltransferase and transpeptidase activities. In the cell, the Lpo proteins must reach through pores in the PG net to interact with their cognate PG synthase. Therefore, it has been proposed that the Lpo-mediated activation of PBPs occurs in response to the properties of the pores in the elastic PG layer to adjust the rate of PG growth with the rate of cell growth (Typas et al., 2010, 2012). Supporting this model, a previous study showed that both outer-membrane-anchored Lpo proteins could be crosslinked to their cognate cytoplasmic membrane-anchored PBP in the cell (Typas et al., 2010). However, in the absence of structural data for full-length LpoA and LpoB, it has remained unclear whether both proteins are long enough to span a distance of at least 110 Å from the outer membrane through the PG layer to interact with their cognate PBP.

The crystal structure of the C-terminal domain of *Haemophilus influenzae* LpoA (LpoA^{C}, residues 257–573) reveals two subdomains that adopt a fold commonly found in periplasmic substrate-binding proteins (Vijayalakshmi et al., 2008). *E. coli* LpoA^{C}

CrossMark
 ©2014 Elsevier Ltd All rights reserved

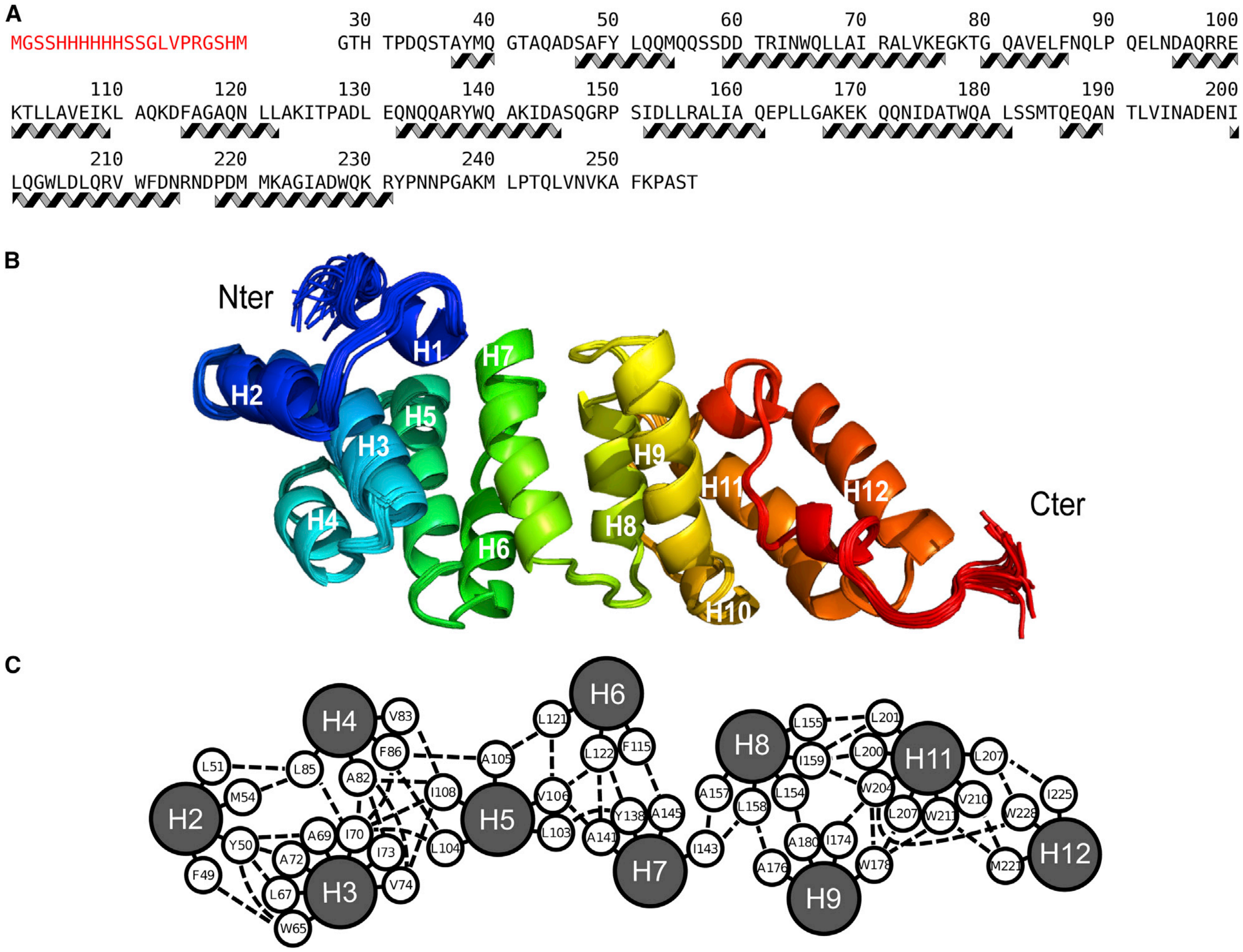

Figure 1. LpoA^N Has a TPR Domain-like Structure

(A) Amino acid sequence of the LpoA^N construct used for structure determination and secondary structure elements (α helices). The residues of the oligohistidine tag are shown in red.

(B) Cartoon representation of the 20 lowest-energy structures of LpoA$^{\mathbf{N}}$, as determined by NMR spectroscopy, emphasizing the spatial arrangement of the 12 α-helices, which are numbered from the N to C terminus.

(C) LpoA^N is stabilized by numerous interhelical hydrophobic contacts. The ten α helices with more than four residues are shown as circles and labeled according to the description in (B). Interhelical hydrophobic contacts are represented with dashed lines, and short plain lines connect each of the residues to its corresponding helix.

See also Figure S1.

has two additional stretches of 67 and 39 amino acids, respectively, that are missing in the *H. influenzae* ortholog. The role of the N-terminal domain (LpoA^N) is not clear, as it does not interact with PBP1A (Typas et al., 2010).

In this work, we present the structure of LpoA^N determined by NMR spectroscopy. LpoA^N is almost completely made of α-helices that form helix-turn-helix motifs similar to tetratricopeptide repeat (TPR) motifs, which are commonly used as a scaffold underpinning protein/protein complexes. We also provide evidence from NMR spectroscopy that LpoA^C contains flexible (unstructured) regions and that the two domains of LpoA are connected by a rather rigid linker. Further biophysical characterization by analytical ultracentrifugation (AUC) and small-angle X-ray scattering (SAXS) showed that full-length LpoA has an elongated molecular shape, supporting the hypothesis that LpoA has the capacity to span the distance from the outer membrane to its cognate cytoplasmic-membrane-anchored PG synthase, PBP1A.

RESULTS

The N-Terminal Domain of LpoA Adopts a TPR-Domain-like Fold

We purified the N-terminal domain of *E. coli* LpoA lacking its signal peptide sequence and outer-membrane lipid anchor (LpoA^N, residues 28–256) fused to an N-terminal oligohistidine tag (Figure 1A and Figure S1A available online). ^{1}H-^{15}N heteronuclear single quantum coherence (^{1}H-^{15}N-HSQC) NMR spectra were recorded from ^{13}C,^{15}N-LpoA^N samples at different temperatures (5°C–50°C) and at different pH values (4.5–7.5), and

 ©2014 Elsevier Ltd All rights reserved

Table 1. Structural Statistics for the Ensemble of 20 NMR Structures of LpoA^N

NMR Distance and Dihedral Constraints	Number/Parameter
Distance Constraints	
Total unambiguous NOE restraints	5,210
Intraresidue	1,826
Interresidue	3,384
Sequential ($\|i-j\| = 1$)	1,141
Medium-range ($\|i-j\| \leq 5$)	1,225
Long-range ($\|i-j\| > 5$)	1,018
Total ambiguous NOE restraints	440
Total Dihedral Angle Restraints	426
Backbone Φ	213
Backbone Ψ	213
Structure Calculation Statistics[a]	
Restraints Violations	
Distance (>0.3 Å, >0.5 Å)	41.95, 4.5
Dihedral (>5°, >6°)	13, 0
Average pairwise root-mean-square deviation (Å)[b]	
Backbone atoms	0.44 ± 0.06
All heavy atoms	0.63 ± 0.05
Ramachandran Analysis[b]	
Residues in most favored regions (%)	85.2
Residues in additional allowed regions (%)	13.6
Residues in generously allowed regions (%)	1.0
Residues in disallowed regions (%)	0.2

[a]Pairwise deviations were calculated among 20 refined structures.
[b]These values were calculated on residues 28–256 (according to numbering in wild-type LpoA).

showed that the global fold of the protein remained unchanged (Figure S1B). For structure determination, NMR spectra were recorded at 50°C and pH 4.5, and chemical shifts were assigned (Jean et al., 2014). The structure of LpoA^N (Figures 1B and 1C) was subsequently determined. Relevant structural and statistical data are reported in Table 1.

LpoA^N is made up of 12 α-helices (H1–H12) of variable length linked together through short turns or rigid loops (Figure 1B). Two additional short stretches of three residues at the C terminus also adopt a characteristic 3_{10}-helix conformation (residues 236–238 and 246– 248). There are numerous interhelical hydrophobic contacts (Figure 1C) favored by the high percentage (24%) of long-chain hydrophobic amino acids and few additional side-chain hydrogen bonds, which explains the stability of the structure over wide pH and temperature ranges (Figure S1B).

LpoA^N has a striking structural similarity to protein domains formed by TPR motifs, as revealed by DALI (Holm and Rosenström, 2010), consistent with the repetition of helix-turn-helix motifs found in these proteins and LpoA^N. The highest score (Z = 9.3) was obtained with a 170-residue stretch of the G protein signaling regulator 2 (Protein Data Bank [PDB] code 3SF4), but both structures had a relatively high root-mean-square deviation (RMSD) value of 5.7 Å. Searching the LpoA^N sequence for canonical TPR repeats (Zeytuni and Zarivach, 2012), TPRpred (Biegert et al., 2006) recognized a TPR sequence pattern only in the I63-A96 fragment comprising H3 and H4, although H4 is shorter than the typical α-helices in TPR motifs. H11/H12 shows a substantial structural similarity to TPR motifs, but was not classified as a TPR motif by TPRpred because long-chain hydrophobic residues replace the conserved alanine residues at positions 8, 20, and 27. The similarity between LpoA^N and TPR domains thus seems limited to the presence of a series of helix-turn-helix motifs with short hydrogen-bonded turns between the two helices (H3/H4, H5/H6, H8/H9, and H11/H12) rather than to the presence of canonical TPR repeats with their characteristic hydrophobic contacts. As in most TPR domains, the individual helices in LpoA^N are organized into a superhelical structure; however, the curvature of this structure is too small to generate the concave and convex surfaces often seen in TPR domains. Instead, LpoA^N shows an overall prolate spheroid shape with a width of ~30 Å and a height of ~70 Å. The protein surface contains three grooves. Two of these grooves localize between the central helices H7 and H8, and the third localizes between H3 and H5. Interestingly, these grooves contain highly conserved residues and are therefore potential interaction sites with proteins or other ligands (Figure S1C).

Full-Length LpoA Has Flexible Regions in the C-Terminal Domain

We next sought to determine the overall molecular shape for full-length LpoA and the relative orientation of the two domains. The N-terminally oligohistidine-tagged protein (Figure S1A) was mostly monomeric (~85%) and contained ~10% dimers. This ratio was similar for all three protein concentrations (6, 2, and 0.2 mg/ml) used in AUC experiments, suggesting that monomers and dimers are not in dynamic exchange. The f/f_{min} ratio of 1.5 and hydrodynamic radius of 4.1 nm, determined from the sedimentation coefficient extrapolated to infinite dilution, s_0 = 4.16 S, are characteristic of an elongated molecular shape and/or a flexible molecule.

Full-length LpoA (see Figure 2 for LpoA^C) has a molecular weight of >70 kDa and therefore is too large for structure determination by NMR spectroscopy. Nonetheless, a ^{1}H-^{15}N band-selective excitation short-transient transverse optimized spectroscopy (^{1}H-^{15}N-BEST-TROSY) NMR spectrum of full-length ^{13}C,^{15}N-LpoA showed ~100 intense and tightly dispersed signals (Figure S2A). The signals seen in LpoA^N were all absent in this spectrum, suggesting that the molecular tumbling of the large anisotropic LpoA molecule prevents the NMR observation of structured (or nonflexible) regions and that only residues in disordered (or flexible) parts of the molecule could be seen. LpoA^N is well structured and therefore most of the signals obtained from full-length LpoA should come from disordered regions in the C-terminal domain. To investigate this possibility, we assigned the detected ^{1}H, ^{13}C, and ^{15}N resonances of a full-length ^{13}C,^{15}N-LpoA sample. HNCACB and BEST-TROSY-(H)N(COCA)NH experiments unambiguously identified stretches of contiguous residues that all reside within the two regions of the *E. coli* LpoA sequence that are absent in *H. influenzae* LpoA (Figure 2). These assignments included backbone resonances from 30 residues between N285 and P351 (region 1) and from 16 residues between S493 and N531 (region 2; Figure S2B). Amino acid types were obtained from Cα and Cβ carbon chemical shifts

 ©2014 Elsevier Ltd All rights reserved

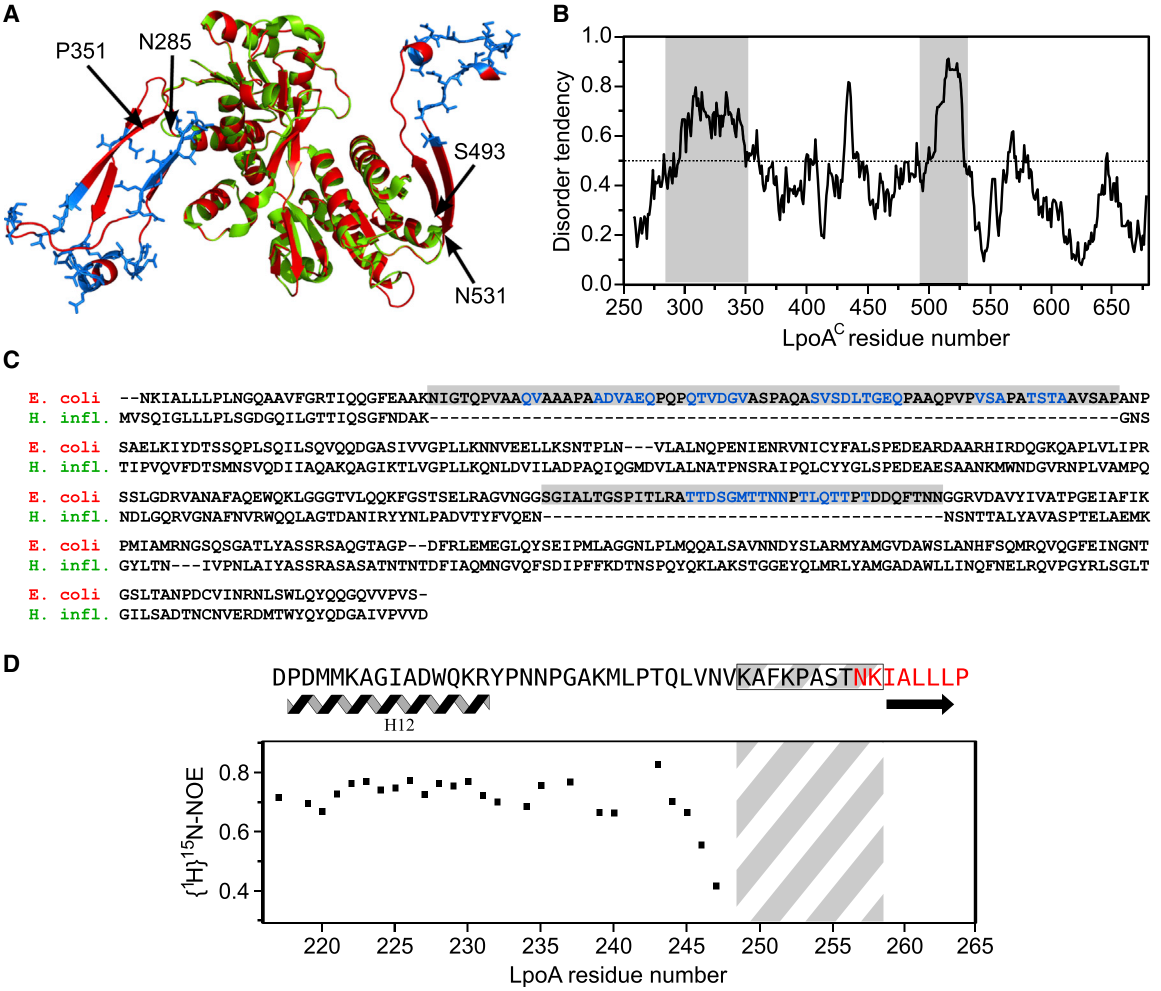

Figure 2. Comparison of LpoA^C from *E. coli* and *H. influenzae*

(A) Superimposition of the X-ray structure of *H. influenzae* LpoA^C (green, PDB code 3CKM) and the structure of *E. coli* LpoA^C predicted by PHYRE (red). Regions 1 (residues N285–P351) and 2 (residues S493–N531) are present in LpoA^C from *E. coli*, but not from *H. influenzae*. Flexible residues for which backbone resonances have been assigned by NMR spectroscopy are sketched as blue sticks.

(B) Disorder in *E. coli* LpoA^C predicted by IUPred. The two main regions absent from the *H. influenzae* LpoA sequence (in gray) are predicted as being mostly unstructured by IUPred (score > 0.5).

(C) Sequence alignment of *H. influenzae* and *E. coli* LpoA^C, where the two *E. coli* inserts are highlighted in gray. NMR-assigned residues are shown in blue.

(D) The linker between LpoA^N and LpoA^C (in gray and white hashes) starts at K249 and ends at K258. The criteria used to define this linker included the structuring of the N-terminal domain as quantified by the $\{^1H\}^{15}N$-NOE measured in LpoA^N (black) and the definition of the first secondary-structure element in the C-terminal domain (red) of LpoA as modeled by PHYRE.

See also Figure S2.

for 36 additional resonances (leaving only eight resonances completely unassigned) and agreed with the expected unassigned amino acids in the two considered regions. In line with these data, regions 1 and 2 were predicted to be unfolded by IUPred (http://iupred.enzim.hu/) with scores higher than 0.5 (Figure 2B).

We subsequently built a structural model of *E. coli* LpoA^C using PHYRE (Kelley and Sternberg, 2009) with the *H. influenzae* LpoA^C structure as a template (Vijayalakshmi et al., 2008; PDB code 3CKM). The superimposition of the *H. influenzae* structure and the *E. coli* model (Figure 2A) emphasizes the presence of extended loops in the flexible regions 1 and 2. To evaluate this model in the light of experimental data, we analyzed chemical shifts from assigned residues in regions 1 and 2, and observed marked dynamics and low secondary-structure propensities at these positions (see Supplemental Experimental Procedures). As a result, region 1 might be more flexible and/or disordered than suggested by the model. In addition to the established loop in region 2, the PHYRE-predicted β sheet (formed by S493-G499 and D525-N531) could not be confirmed by NMR spectroscopy due to incomplete sequence-specific assignments.

LpoA Has a Limited Interdomain Flexibility

If there is a highly flexible linker between the N- and C-terminal domains, full-length $^{13}C,^{15}N$-LpoA should give rise to NMR

 ©2014 Elsevier Ltd All rights reserved

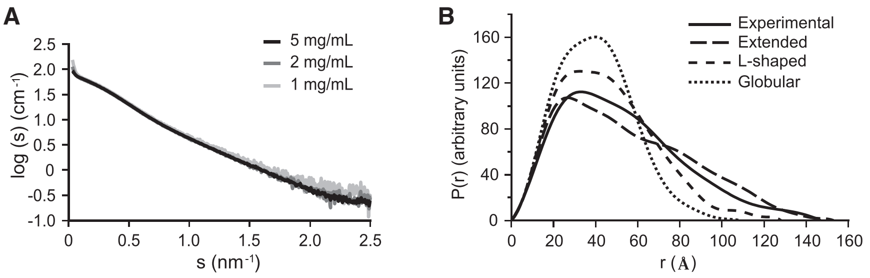

Figure 3. Full-Length LpoA Has an Extended Structure

(A) SAXS curves of LpoA at concentrations of 1, 2, and 5 mg/ml.

(B) Experimental distance distribution function, P(r), calculated from SAXS data collected on a 5 mg/ml ^{15}N-LpoA sample (black) and theoretical P(r) function calculated for molecular models with three different, arbitrarily chosen orientations of the N- and C-terminal domains (....., globular model; ----, L-shaped model; – – –, extended model; see also Figure S3C). The theoretical R_g values extracted from these curves are 3.11 nm for the globular model, 3.58 nm for the L-shaped model, and 4.44 nm for the extended model. The experimental R_g value, 4.22 ± 0.01 nm, fits best to the extended model.

signals corresponding to a sum of the signals observed for the separate domains. However, characteristic LpoA^N resonances remained undetected in this sample after an increase of the signal-to-noise ratio by a factor of 2. Only the virtually complete perdeuteration of full-length LpoA and the spectroscopic advantage of the TROSY effect allowed the detection of LpoA^N and LpoA^C amide resonances (Figure S2C). The structured regions of full-length LpoA, comprising most of the N- and C-terminal domains, thus behave as a highly anisotropic single structural entity without significant flexibility between the domains. This hypothesis was further confirmed by the similar heteronuclear multiple quantum coherence (HMQC) versus HSQC intensity ratios (Tugarinov et al., 2003) for ^{1}H-^{13}C correlations of alanine methyl groups in a U-[^{2}H, ^{12}C, ^{15}N], Val-[^{13}C^{1}H$_3$]$^{pro\text{-}S}$, Ala-[^{13}C^{1}H$_3$]-LpoA sample (Figure S2D), indicating a correlated rotational motion of the two domains. A strong, rather rigid interaction between LpoA$^{\mathbf{N}}$ and LpoA$^{\mathbf{C}}$ also agrees with the low disorder score of <0.38 calculated by IUPred for the linker region.

Full-Length LpoA Has an Elongated Molecular Shape

We used SAXS to determine the overall molecular shape of full-length LpoA (Figure 3). The estimated molecular weights of LpoA by SAXS (66.7 kDa, 64.9 kDa, and 68.2 kDa) were similar for each concentration tested (5, 2, and 1 mg/ml, respectively), showing that the scattering intensities recorded came mostly from the monomeric form of the protein and confirming the largely monomeric and monodisperse behavior of LpoA (Figure 3A). The distance distribution function P(r) was calculated from the SAXS data recorded at the highest protein concentration (Figure 3B) and yielded a radius of gyration (R_g) of 4.22 ± 0.01 nm, which is similar to the R_g of 4.17 ± 0.02 nm calculated by the Guinier approximation (Figure S3A).

We next built hypothetical models of full-length LpoA with different relative orientations of the N- and C-terminal domains using the structure of LpoA^N and the previously built *E. coli* LpoA^C homology model (Figure S3C) (Svergun, 1992), and calculated their theoretical P(r) and R_g values. The elongated putative LpoA model had a theoretical R_g that was close to the experimental value (4.44 nm versus 4.22 nm), whereas the putative globular and L-shaped models had lower theoretical R_g values (3.11 nm and 3.58 nm, respectively). The observed P(r) is typical of an oblate shape and is similar to the curve calculated for the elongated model (Figure 3B). The calculated D_{max} (the longest distance between two points in the protein) of 146.8 Å is also consistent with an elongated arrangement of the two domains, as LpoA^N and LpoA^C have domain lengths of ~70 Å and ~60 Å, respectively. Thus, although the presence of extended flexible regions (regions 1 and 2) in the C-terminal domain (Figure S3B) prevented us from building a higher-resolution model of LpoA, the SAXS data are consistent with an elongated structure.

DISCUSSION

In this work, we determined the structure of the N-terminal domain of LpoA and showed that it adopts a TPR-like fold. Proteins containing TPR domains (or modules) are ubiquitous and are present in organisms belonging to all kingdoms of life (Zeytuni and Zarivach, 2012). It is generally accepted that TPR modules do not exhibit an enzymatic activity, but rather serve as interaction modules in multiprotein assemblies or for protein dimerization (Allan and Ratajczak, 2011). For example, the TPR domain of BamD, an essential component of the outer-membrane β-barrel assembly machinery in Gram-negative bacteria, interacts with the N-terminal, unstructured region of BamC (Kim et al., 2011; Sandoval et al., 2011).

The analysis of 63 unique LpoA^N sequences revealed two conserved patches on the protein surface, which could represent potential interaction sites (Figure S1C). PBP1A interacts with LpoA^C, but not with LpoA^N (Typas et al., 2010), and LpoA and LpoA^N exist predominantly as monomers, suggesting that the TPR-like motifs do not promote self-interaction. Rather, LpoA^N may interact with one or more as yet unknown protein(s). Studies to identify the interaction partners of LpoA^N are currently under way.

Full-length LpoA yielded NMR signals that originate from two flexible regions in the C-terminal domain. Other species, such as *H. influenzae*, either lack these stretches or have different stretches at the same or a different position in the LpoA^C sequence (Vijayalakshmi et al., 2008). The functions of the two flexible regions in *E. coli* LpoA^C are currently unknown. It is possible that they fold into rigid structures under certain conditions, perhaps during the formation of new protein-protein interactions.

The NMR analysis of full-length LpoA also indicated that LpoA^N is not capable of moving independently of LpoA^C. Instead, both domains must be connected rather rigidly, consistent with the absence of any flexible stretch in this region. NMR,

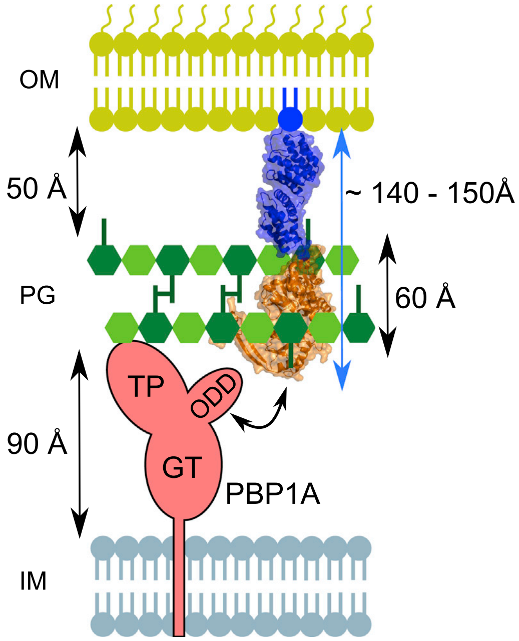

Figure 4. Schematic Representation of PBP1A Activation by LpoA
The N-terminal domain of LpoA (blue) anchors to the outer membrane, whereas the C-terminal domain (orange) interacts with the outer-membrane PBP1A docking domain (Typas et al., 2010). LpoA has an estimated total width of ~30 Å and length of ~145 Å. These dimensions should enable the protein to reach PBP1A through the periplasm and cross the ~60-Å-thick PG layer, which has ~40- to 60-Å-wide pores (Demchick and Koch, 1996). TP, transpeptidase domain; GT, glycosyltransferase domain; IM, inner membrane; OM, outer membrane.

AUC, and SAXS measurements support an elongated molecular shape of LpoA with a length of ~145 Å. With this length, LpoA would be capable of spanning from the outer membrane through the periplasm and PG layer to reach a bifunctional PG synthase, such as PBP1A, whose TPase domain is up to ~100 Å away from the cytoplasmic membrane (Sung et al., 2009; Figure 4). The PG layer is elastic and has pores with a diameter of ~40 Å in the relaxed state, allowing the diffusion of globular proteins of up to 25 kDa (Demchick and Koch, 1996; Vollmer and Höltje, 2004). The pores in stretched PG may have a diameter of ~60 Å and allow the diffusion of proteins of up to ~100 kDa (Demchick and Koch, 1996; Vázquez-Laslop et al., 2001). Hence, LpoA with a width of ~30 Å should be able to penetrate the pores present in PG. Therefore, our data on the molecular dimensions and shape of LpoA are consistent with the observed crosslinking of LpoA with PBP1A in intact cells (Typas et al., 2010).

The synthesis of new PG and its incorporation into the existing cell wall in growing and dividing bacteria is a well-regulated process, ensuring that the growth of all cell envelope layers is coordinated with cell growth. We are only beginning to understand the complexity of these processes and still lack insights into crucial molecular details of PG growth. PG synthases engage in multiple interactions with other PG enzymes, and the cytoskeletal proteins FtsZ and MreB with associated proteins are essential to guide PG growth (Typas et al., 2012). Presumably, these proteins form large multiprotein complexes for PG synthesis during cell elongation and division, called the elongasome and divisome, respectively (Szwedziak and Löwe, 2013). Bifunctional PG synthases with both glycosyltransferase and transpeptidase activities are part of these complexes and play a major role in PG growth, but only few structures of these important enzymes are known (Han et al., 2011; Lovering et al., 2007; Sung et al., 2009). Hence, we need more structural data on PG enzymes and their interacting proteins. In this report, we have presented data on LpoA that form the structural basis for understanding the activation of PG synthases by outer-membrane proteins.

EXPERIMENTAL PROCEDURES

Protein Purification

BL21(DE3) strains harboring pET28LpoA or pET28LpoA^N (Typas et al., 2010) were used to produce soluble full-length LpoA or LpoA^N (lipid anchor replaced by an oligohistidine tag) with different isotopic labeling schemes (details in Supplemental Experimental Procedures).

NMR Spectroscopy

NMR data were collected on a 2.5 mM [^{13}C, ^{15}N]-LpoA^N sample in 100 mM sodium acetate buffer, pH 4.5, containing 10% D_2O at 323 K. Backbone and side-chain resonances were assigned as previously described (Jean et al., 2014). For the assignment of backbone resonances of flexible regions of full-length LpoA, a 3D HNCACB and a 3D BEST-TROSY-(H)N(COCA)NH (Solyom et al., 2013) were collected at 293 K on a 0.75 mM [^{13}C, ^{15}N]-LpoA sample in 50 mM HEPES, 100 mM NaCl, pH 6.5, containing 10% D_2O (buffer I), on Bruker spectrometers operating at 700 and 950 MHz ^{1}H NMR frequency, equipped with triple ^{1}H, ^{15}N, ^{13}C-resonance cryoprobes. 2D ^{1}H-^{15}N-BEST-TROSY and 2D-methyl-HMQC/HSQC (Tugarinov et al., 2003) spectra were recorded on the 950-MHz Bruker spectrometer on a 72 μM U-[^{2}H, ^{12}C, ^{15}N], Val-[^{13}C^{1}H$_3$]$^{pro\text{-}S}$, Ala-[^{13}C^{1}H$_3$]-LpoA, or 297 μM U-[^{2}H, ^{12}C, ^{15}N]-LpoA sample in buffer I at 293 K. Details regarding the data analysis are reported in Supplemental Experimental Procedures.

Extraction of Structural Restraints and Structure Calculation

Distance restraints from 3D ^{15}N-NOESY-HSQC, and 3D aliphatic, aromatic ^{13}C-NOESY-HSQC experiments were obtained by using UNIO'10 version 2.0.2 (Guerry and Herrmann, 2012). Additional distance constraints were extracted from peak volumes from a methyl-^{13}C-NOESY-HSQC experiment assigned manually. TALOS+ was used to determine phi/psi dihedral angle restraints from chemical shifts (Shen et al., 2009). The structure of LpoA^N was calculated with Aria 2.3.1 (Rieping et al., 2007) and 100 structures were calculated in each iteration, except in the last cycle when 700 structures were calculated. The 20 lowest-energy structures underwent explicit water refinement. The structures were visualized graphically and all figures were made with the Pymol Molecular Graphics System, version 1.5.0.4 (Schrödinger, LLC).

AUC

Sedimentation velocity experiments were performed on an analytical ultracentrifuge XLI (Beckman Coulter) operating at 293 K with a rotor speed of 42,000 rpm. Three ^{15}N-labeled LpoA samples (6, 2, and 0.2 mg/ml) in 50 mM HEPES, 100 mM NaCl, pH 6.5, were loaded on double-sector centerpieces in the Anti-50 rotor with optical path lengths of 1.5, 3 and 12 mm, respectively (Nanolytics). The data were acquired at 280 nm using interference optics and analyzed with SEDFIT 14.1 (Schuck, 2000).

SAXS

SAXS data were collected on beamline BM29 at the European Synchrotron Radiation Facility (Grenoble, France). Scattering data were collected on three ^{15}N-LpoA samples with concentrations of 5, 2, and 1 mg/ml, in the same buffer conditions as in the AUC experiments. Each sample was positioned at 2.87 m from a Pilatus detector and ten frames of 1 s were recorded at a

 ©2014 Elsevier Ltd All rights reserved

wavelength of 0.99 Å. After normalization to the intensity of the transmitted beam, frames were merged for each sample. Subtraction of the buffer's contribution to the scattering and further processing steps were performed with PRIMUS from the ATSAS 2.5.1 program package (Svergun, 1992). The radius of gyration, R_g, forward scattering intensity, I(0), maximum particle dimension, D_{max}, and distance distribution function, P(r) were evaluated with GNOM. The R_g was also estimated using the Guinier approximation in the Autorg software (Petoukhov et al., 2007). The NMR structure of $LpoA^N$ and a model structure of *E. coli* $LpoA^C$, predicted by PHYRE (Kelley and Sternberg, 2009) from its homolog in *H. influenzae*, were used to calculate the R_g and P(r). Globular, L-shaped, and elongated models of full-length LpoA were created using different dihedral angles in the linker. Scattering curves were then simulated on these models by CRYSOL. Their respective distance distribution function and the parameters R_g and D_{max} were calculated by GNOM.

ACCESSION NUMBERS

The coordinates of 20 structures and chemical shifts of $LpoA^N$ have been deposited in the Protein Data Bank and the BioMagResBank under accession codes 2MHK and 18853, respectively.

SUPPLEMENTAL INFORMATION

Supplemental Information includes Supplemental Experimental Procedures and three figures and can be found with this article online at http://dx.doi.org/10.1016/j.str.2014.04.017.

ACKNOWLEDGMENTS

We thank L. Signor, A. Le Roy, C. Ebel, and the staff of the ESRF and EMBL-Grenoble for assistance, support, and access to the Mass Spectrometry Facility, the Analytical Ultracentrifugation Platform, and beamline BM29. We thank J. Boisbouvier, B. Brutscher, D. Marion, and P. Schanda for stimulating discussions, and NMR-Bio for the kind gift of deuterated alanine and acetolactate precursors. W.V. and R.J.L. were supported by the BBSRC (BB/I020012/1). W.V. was also supported by the EC DIVINOCELL (HEALTH-F3-2009-223431). A.D. was supported by an EMBO long-term fellowship and N.L.J. was supported by a PhD fellowship (CFR) from the CEA. This work used the platforms of the Grenoble Instruct Centre (ISBG; UMS 3518 CNRS-CEA-UJF-EMBL) with support from FRISBI (ANR-10-INSB-05-02) and GRAL (ANR-10-LABX-49-01) within the Grenoble Partnership for Structural Biology (PSB). Financial support was also provided by the IR-RMN-THC FR3050 CNRS to conduct the NMR research.

Received: December 3, 2013
Revised: April 25, 2014
Accepted: April 28, 2014
Published: June 19, 2014

REFERENCES

Allan, R.K., and Ratajczak, T. (2011). Versatile TPR domains accommodate different modes of target protein recognition and function. Cell Stress Chaperones *16*, 353–367.

Banzhaf, M., van den Berg van Saparoea, B., Terrak, M., Fraipont, C., Egan, A., Philippe, J., Zapun, A., Breukink, E., Nguyen-Distèche, M., den Blaauwen, T., and Vollmer, W. (2012). Cooperativity of peptidoglycan synthases active in bacterial cell elongation. Mol. Microbiol. *85*, 179–194.

Bertsche, U., Breukink, E., Kast, T., and Vollmer, W. (2005). *In vitro* murein peptidoglycan synthesis by dimers of the bifunctional transglycosylase-transpeptidase PBP1B from *Escherichia coli*. J. Biol. Chem. *280*, 38096–38101.

Biegert, A., Mayer, C., Remmert, M., Söding, J., and Lupas, A.N. (2006). The MPI Bioinformatics Toolkit for protein sequence analysis. Nucleic Acids Res. *34*, W335–W339.

Born, P., Breukink, E., and Vollmer, W. (2006). In vitro synthesis of cross-linked murein and its attachment to sacculi by PBP1A from *Escherichia coli*. J. Biol. Chem. *281*, 26985–26993.

Demchick, P., and Koch, A.L. (1996). The permeability of the wall fabric of *Escherichia coli* and *Bacillus subtilis*. J. Bacteriol. *178*, 768–773.

Guerry, P., and Herrmann, T. (2012). Comprehensive automation for NMR structure determination of proteins. Methods Mol. Biol. *831*, 429–451.

Han, S., Caspers, N., Zaniewski, R.P., Lacey, B.M., Tomaras, A.P., Feng, X., Geoghegan, K.F., and Shanmugasundaram, V. (2011). Distinctive attributes of β-lactam target proteins in Acinetobacter baumannii relevant to development of new antibiotics. J. Am. Chem. Soc. *133*, 20536–20545.

Holm, L., and Rosenström, P. (2010). Dali server: conservation mapping in 3D. Nucleic Acids Res. *38*, W545–W549.

Jean, N.L., Bougault, C., Derouaux, A., Callens, G., Vollmer, W., and Simorre, J.P. (2014). Backbone and side-chain ^1H, ^{13}C, and ^{15}N NMR assignments of the N-terminal domain of *Escherichia coli* LpoA. Biomol. NMR Assign. Published online February 4, 2014. http://dx.doi.org/10.1007/s12104-014-9546-2.

Kelley, L.A., and Sternberg, M.J. (2009). Protein structure prediction on the Web: a case study using the Phyre server. Nat. Protoc. *4*, 363–371.

Kim, K.H., Aulakh, S., and Paetzel, M. (2011). Crystal structure of β-barrel assembly machinery BamCD protein complex. J. Biol. Chem. *286*, 39116–39121.

Lovering, A.L., de Castro, L.H., Lim, D., and Strynadka, N.C. (2007). Structural insight into the transglycosylation step of bacterial cell-wall biosynthesis. Science *315*, 1402–1405.

Paradis-Bleau, C., Markovski, M., Uehara, T., Lupoli, T.J., Walker, S., Kahne, D.E., and Bernhardt, T.G. (2010). Lipoprotein cofactors located in the outer membrane activate bacterial cell wall polymerases. Cell *143*, 1110–1120.

Petoukhov, M.V., Konarev, P.V., Kikhney, A.G., and Svergun, D.I. (2007). ATSAS 2.1—towards automated and web-supported small-angle scattering data analysis. J. Appl. Cryst. *40*, 223–228.

Rieping, W., Habeck, M., Bardiaux, B., Bernard, A., Malliavin, T.E., and Nilges, M. (2007). ARIA2: automated NOE assignment and data integration in NMR structure calculation. Bioinformatics *23*, 381–382.

Sandoval, C.M., Baker, S.L., Jansen, K., Metzner, S.I., and Sousa, M.C. (2011). Crystal structure of BamD: an essential component of the β-Barrel assembly machinery of Gram-negative bacteria. J. Mol. Biol. *409*, 348–357.

Schuck, P. (2000). Size-distribution analysis of macromolecules by sedimentation velocity ultracentrifugation and lamm equation modeling. Biophys. J. *78*, 1606–1619.

Shen, Y., Delaglio, F., Cornilescu, G., and Bax, A. (2009). TALOS+: a hybrid method for predicting protein backbone torsion angles from NMR chemical shifts. J. Biomol. NMR *44*, 213–223.

Solyom, Z., Schwarten, M., Geist, L., Konrat, R., Willbold, D., and Brutscher, B. (2013). BEST-TROSY experiments for time-efficient sequential resonance assignment of large disordered proteins. J. Biomol. NMR *55*, 311–321.

Sung, M.T., Lai, Y.T., Huang, C.Y., Chou, L.Y., Shih, H.W., Cheng, W.C., Wong, C.H., and Ma, C. (2009). Crystal structure of the membrane-bound bifunctional transglycosylase PBP1b from *Escherichia coli*. Proc. Natl. Acad. Sci. USA *106*, 8824–8829.

Svergun, D.I. (1992). Determination of the regularization parameter in indirect-transform methods using perceptual criteria. J. Appl. Cryst. *25*, 495–503.

Szwedziak, P., and Löwe, J. (2013). Do the divisome and elongasome share a common evolutionary past? Curr. Opin. Microbiol. *16*, 745–751.

Tugarinov, V., Hwang, P.M., Ollerenshaw, J.E., and Kay, L.E. (2003). Cross-correlated relaxation enhanced 1H-13C NMR spectroscopy of methyl groups in very high molecular weight proteins and protein complexes. J. Am. Chem. Soc. *125*, 10420–10428.

Typas, A., Banzhaf, M., van den Berg van Saparoea, B., Verheul, J., Biboy, J., Nichols, R.J., Zietek, M., Beilharz, K., Kannenberg, K., von Rechenberg, M., et al. (2010). Regulation of peptidoglycan synthesis by outer-membrane proteins. Cell *143*, 1097–1109.

 ©2014 Elsevier Ltd All rights reserved

Typas, A., Banzhaf, M., Gross, C.A., and Vollmer, W. (2012). From the regulation of peptidoglycan synthesis to bacterial growth and morphology. Nat. Rev. Microbiol. *10*, 123–136.

Vázquez-Laslop, N., Lee, H., Hu, R., and Neyfakh, A.A. (2001). Molecular sieve mechanism of selective release of cytoplasmic proteins by osmotically shocked *Escherichia coli*. J. Bacteriol. *183*, 2399–2404.

Vijayalakshmi, J., Akerley, B.J., and Saper, M.A. (2008). Structure of YraM, a protein essential for growth of *Haemophilus influenzae*. Proteins *73*, 204–217.

Vollmer, W., and Höltje, J.-V. (2004). The architecture of the murein (peptidoglycan) in Gram-negative bacteria: vertical scaffold or horizontal layer(s)? J. Bacteriol. *186*, 5978–5987.

Vollmer, W., Blanot, D., and de Pedro, M.A. (2008). Peptidoglycan structure and architecture. FEMS Microbiol. Rev. *32*, 149–167.

Zeytuni, N., and Zarivach, R. (2012). Structural and functional discussion of the tetra-trico-peptide repeat, a protein interaction module. Structure *20*, 397–405.

 ©2014 Elsevier Ltd All rights reserved

Structure, Volume *22*

Supplemental Information

Elongated Structure of the Outer-Membrane Activator of Peptidoglycan Synthesis LpoA: Implications for PBP1A Stimulation

Nicolas L. Jean, Catherine M. Bougault, Adam Lodge, Adeline Derouaux, Gilles Callens, Alexander J.F. Egan, Isabel Ayala, Richard J. Lewis, Waldemar Vollmer, and Jean-Pierre Simorre

SUPPLEMENTAL INFORMATION

The elongated structure of the outer-membrane activator of peptidoglycan synthesis LpoA: implications in PBP1A-stimulation

Nicolas L. Jean, Catherine M. Bougault, Adam Lodge, Adeline Derouaux, Gilles Callens, Alexander J. F. Egan, Isabel Ayala, Richard J. Lewis, Waldemar Vollmer,* Jean-Pierre Simorre*

Inventory of Supplemental Information

Supplemental Experimental Procedures **2**

Expression and purification of LpoA versions. 2

Secondary structure propensities in the flexible regions 1 and 2 of full-length LpoA 4

NMR data processing and analysis. 5

Supplemental References **5**

Supplemental Figures

Figure S1, related to Figure 1 **7**

Characterization of $LpoA^N$ and full-length LpoA in different conditions.

Figure S2, related to Figure 2 **9**

Characteristic spectroscopic signatures of $LpoA^N$ and full-length LpoA.

Figure S3, related to Figure 3 **12**

Guignier and Kratky plots calculated from SAXS data measured on a 5 mg/mL LpoA sample.

Supplemental Experimental Procedures

Expression and purification of LpoA versions.

Unlabeled LpoA and LpoA^N. BL21(DE3) strains harboring plasmids pET28LpoA or pET28LpoA^N were used to purify soluble full-length LpoA or LpoA^N (lipid anchor replaced by an oligohistidine tag). Three liters of LB medium (10 g/L tryptone, 5 g/L yeast extract, 10 g/L NaCl) containing 50 µg/mL kanamycin were inoculated 1 in 50 with a corresponding overnight culture and incubated at 37°C until the OD_{578} reached 0.4 – 0.6. Overexpression of recombinant *lpoA* genes was induced by the addition of 1 mM IPTG and further incubation for 3 h at 30°C. Cells were harvested by centrifugation (10,000 × g, 15 min, 4°C) and the pellet was resuspended in 40 mL of buffer I (25 mM Tris/HCl, 10 mM $MgCl_2$, 500 mM NaCl, 20 mM imidazole, 10% glycerol, pH 7.5). DNase, protease inhibitor cocktail (Sigma) (1 to 1000 dilution) and 100 µM phenylmethylsulfonylfluoride (PMSF) was added before cells were disrupted by sonication using a Branson digital sonicator. The lysate was centrifuged (130,000 × g, 60 min, 4°C) and the supernatant was applied at a 1 mL/min flow rate to a 5 mL HisTrap HP column (GE Healthcare) attached to an ÄKTA PrimePlus (GE Healthcare) FPLC. The column was washed with 4 volumes of buffer I before step-wise elution of bound proteins with buffer II (25 mM Tris/HCl, 10 mM $MgCl_2$, 500 mM NaCl, 400 mM imidazole, 10% glycerol, pH 7.5). If a second purification step was required, proteins were dialyzed against IEX buffer A (20 mM Tris/HCl, pH 8.0) and applied to a 5 mL HiTrap Q HP column (GE healthcare) at a flow rate of 0.5 mL/min using an ÄKTA PrimePlus FPLC system. The column was washed with 85% IEX buffer A and 15% IEX buffer B (20 mM Tris/HCl, 500 mM NaCl, pH 8.0) for 10 column volumes at 2 mL/min before a linear gradient from 15% to 100% B over 150 mL was applied at 2 mL/min. The eluted LpoA protein was pooled and concentrated to 4 – 5 mL for application to a Superdex200 HiLoad 16/600 column at 1 mL/min for size exclusion chromatography in a buffer containing 25 mM Tris/HCl, 10 mM $MgCl_2$, 500 mM NaCl, 10% glycerol at pH 7.5. In the case of LpoA^N the second purification step (ion exchange chromatography) was omitted.

U-^{15}N-LpoA, U-[^{13}C, ^{15}N]-LpoA and U-[^{13}C, ^{15}N]-LpoA^{N}. For the production of ^{15}N- or ^{13}C,^{15}N-isotopically labeled versions, 2 L M9 growth medium (5.29 g/L Na_2HPO_4, 3 g/L KH_2PO_4, 0.5 g/L NaCl, 1 g/L NH_4Cl, 2 mM thiamine, 1 mM $MgSO_4$, 0.1 mM $CaCl_2$, 0.3% glucose, pH 6.8 – 7.2) containing 50 µg/mL kanamycin were used. For single labeled protein [^{15}N]-NH_4Cl was used, for double labeled protein both [^{15}N]-NH_4Cl and [^{13}C]-glucose were used (Cambridge isotope laboratories Inc, USA). Cells were grown overnight in 100 mL M9 medium per liter of final culture, harvested by centrifugation (3,000 × g, 20 min, RT) and resuspended in 2 × 1 mL of fresh M9 medium which was used to inoculate the remaining 900 mL M9 medium containing the desired isotopically labelled compound(s). Purification proceeded by affinity and gel filtration chromatography as described above.

Perdeuterated LpoA samples. The U-[^{2}H, ^{15}N, ^{12}C], Val-[2,3-^{2}H$_2$; 1,2,3-^{12}C$_3$; [^{12}C^{2}H$_3$]$^{pro\text{-}R}$/[^{13}C^{1}H$_3$]$^{pro\text{-}S}$], Ala-[2-^{12}C^{2}H; 3-^{13}C^{1}H$_3$]-LpoA sample (labeled in short U-[^{2}H, ^{15}N, ^{12}C], Val-[^{13}C^{1}H$_3$]$^{pro\text{-}S}$], Ala-[^{13}C^{1}H$_3$]-LpoA) was prepared according to our published protocols (Ayala et al., 2009; Mas et al., 2013). *E. coli* BL21(DE3) carrying the LpoA plasmid were progressively adapted in three stages over 24 h to an enriched M9/D_2O medium containing 5.3 g/L Na_2HPO_4, 3 g/L KH_2PO_4, 0.5 g/L NaCl, 1 g/L ^{15}NH_4Cl, 1 mM thiamine, 1 mM $MgSO_4$, 0.1 mM $CaCl_2$, 50 µM $ZnSO_4$, 100 µM $FeCl_3$, 30 mg/L kanamycin, a vitamin cocktail, and 2 g/L D-glucose-d$_7$ (^{12}C$_6$^{1}H$_5$^{2}H$_7$O$_6$, Isotec) at pH 6.8 – 7.2. Two 500 mL cultures in enriched M9/D_2O medium were inoculated and grown at 37°C. When the OD_{600} reached 0.6 to 0.8, solutions of [2-^{12}C^{2}H; 3-^{13}C^{1}H$_3$]-L-alanine (NMR-Bio), *pro-S* acetolactate-^{13}C (2-hydroxy-2-[^{13}C]methyl-3-oxo-4,4,4-tri-[^{2}H]butanoate, NMR-Bio) and L-leucine-d$_{10}$ (Sigma-Aldrich) in D_2O were added to final concentrations of 500 mg/L, 240 mg/L and 30 mg/L, respectively. After one hour, protein expression was induced by the addition of 1 mM IPTG and the culture was incubated at 20°C overnight. Cells were harvested by centrifugation (5,000 × g, 10 min, 4°C) and the pellet was resuspended in 40 mL of buffer I (25 mM Tris/HCl, 100 mM NaCl, pH 7.5). Purification proceeded through affinity and gel filtration chromatography steps as described above. To prevent LpoA sample degradation, purification steps were led at 4°C in

presence of Complete^{TM} protease inhibitor cocktail (Roche).

The U-[^{2}H, ^{15}N, ^{12}C]-LpoA sample was prepared according to the protocol described by Rasia et al. in 2009. *E. coli* BL21(DE3) bacterial cells harboring the LpoA plasmid were grown in M9/H_2O medium containing 10 g/L $Na_2HPO_4.7H_2O$, 3 g/L KH_2PO_4, 0.5 g/L NaCl, 1 g/L $^{15}NH_4Cl$, 1 mM thiamine, 1 mM $MgSO_4$, 0.1 mM $CaCl_2$, 50 μM $ZnSO_4$, 100 μM $FeCl_3$, 30 mg/L kanamycin, a vitamin cocktail, and 2 g/L D-glucose-d$_7$ ($^{12}C_6{}^1H_5{}^2H_7O_6$, Isotec) at 37°C. When the OD_{600} reached 0.5 to 0.6, the medium was supplemented with 3 g/L -[^{2}H, ^{15}N, ^{12}C]-ISOGRO^{TM} powder (Isotec). After one hour, 1 mM IPTG was added and the culture was incubated at 20°C overnight. The purification protocol was identical to the one described just above. Due to the presence of H_2O instead of D_2O in the culture medium, all the water exchangeable nuclei remained protonated, in contrast to the previous sample.

Both perdeuterated protein samples were characterized by gel electrophoresis and the HPLC profile from the gel filtration chromatography and showed identical properties as the U-[^{1}H, ^{13}C, ^{15}N] sample.

Secondary structure propensities in the flexible regions 1 and 2 of full-length LpoA.

The ^{1}H, ^{15}N and ^{13}C chemical shifts from assigned residues in the flexible regions 1 (N285 – P351) and 2 (S493 – N531) of full-length LpoA have been analyzed to determine secondary structure propensity. Chemical shift differences with respect to random coil values corrected with sequence-dependent effects (Schwarzinger et al., 2001) failed to identify secondary structure elements. The SSP software (Marsh et al., 2006) computed 10.5% and 1.4% β-structure and 0.1% and 1.0% α-helical propensity for regions 1 and 2, respectively. MICS (Shen and Bax, 2012) and TALOS+ (Shen et al., 2009) showed marked dynamics for regions 1 and 2 with S^2 values of 0.449 ± 0.091 and 0.458 ± 0.105. These predictions are consistent with the either positive and below 0.2 or negative {^{1}H}-^{15}N-NOE values measured for the sequence specifically assigned residues. The latter programs predicted a higher uniformly distributed β-structure propensity in regions 1 and 2

(28% and 24%, respectively). Delta2D (Camilloni et al., 2012), developed specifically for the analysis of disordered proteins, gave 6.1% and 7.5% β-structure and 87.5% and 83.8% coil propensity for regions 1 and 2, respectively. Consequently, regions 1 and 2 appear disordered or dynamic, at least for the assigned residues, which include a large portion of region 1 and the loop region of region 2. The secondary structure in these regions cannot be determined conclusively from the different programs used because of the discrepancies of their outputs, which may in part be correlated to the incompleteness of the specific resonance assignments for these regions.

NMR data processing and analysis.

NMR data were processed with NMRPipe (Delaglio et al., 1995) and analyzed using the CcpNmr Analysis 2.2 software (Vranken et al., 2005). To estimate correlation times of the N-terminal and C-terminal domain of full-length LpoA, the intensity of ^{1}H-^{13}C correlations of methyl groups for alanine residues in the HMQC (I_{HMQC}) and HSQC (I_{HSQC}) spectra were determined, after fitting of the individual lineshapes with a Gaussian function within the CcpNmr software. Intensity ratios I_{HMQC}/I_{HSQC} were calculated for each individual methyl group. Similar ratios (average value 1.3) were obtained for resolved alanine correlations arising from the N-terminal and C-terminal domains, suggesting a similar correlation time for the two domains. These ratios were drastically different and showed a systematic enhancement with respect to ratios calculated for residues arising from the flexible regions 1 and 2 in the C-terminal domain.

Supplemental References

Ashkenazy, H., Erez, E., Martz, E., Pupko, T., and Ben-Tal, N. (2010). ConSurf 2010: calculating evolutionary conservation in sequence and structure of proteins and nucleic acids. Nucleic Acids Res. *38*, W529-W533.

Ayala, I., Sounier, R., Usé, N., Gans, P., and Boisbouvier, J. (2009). An efficient protocol for the complete incorporation of methyl-protonated alanine in perdeuterated protein. J Biomol NMR, *43*, 111-119.

Camilloni, C., De Simone, A., Vranken, W., and Vendruscolo,, M. (2012). Determination of secondary structure populations in disordered states of proteins using NMR chemical shifts. Biochemistry *51*, 2224-2231.

Delaglio, F., Grzesiek, S., Vuister, G.W., Zhu, G., Pfeifer, J., and Bax, A. (1995). NMRPipe: a multidimensional spectral processing system based on UNIX pipes. J Biomol NMR *6*, 277-293.

Marsh, J.A., Singh, V.K., Jia, Z. and Forman-Kay, J.D. (2006). Sensitivity of secondary structure propensities to sequence differences between α- and γ-synuclein: Implications for fibrillation. Protein Science *15*, 2795–2804.

Mas, G., Crublet, E., Hamelin, O., Gans, P., and Boisbouvier, J. (2013). Specific labeling and assignment strategies of valine methyl groups for NMR studies of high molecular weight proteins. J Biomol NMR, *57*, 251-262.

Rasia, R.M., Noirclerc-Savoye, M., Bologna, N.G., Gallet, B., Plevin, M.J., Blanchard, L., Palatnik, J.F., Brutscher, B., Vernet, T., and Boisbouvier, J. (2009). Parallel screening and optimization of protein constructs for structural studies. Protein Sci *18*, 434-439.

Shen, Y., Delaglio, F., Cornilescu, G., and Bax, A. (2009). TALOS+: a hybrid method for predicting protein backbone torsion angles from NMR chemical shifts. J Biomol NMR *44*, 213-223.

Shen, Y., and Bax, A. (2012). Identification of helix capping and beta-turn motifs from NMR chemical shifts. J. Biomol. NMR *52*, 211-232.

Schwarzinger, S., Kroon, G.J.A., Foss, T.R., Chung J., Wright, P.E., and Dyson, H.J. (2001). Sequence-dependent correction of random coil NMR chemical shifts. J Am Chem Soc *123*, 2970-2978.

Vranken, W.F., Vriend, G., and Vuister, G.W. (2012). CING: an integrated residue-based structure validation program suite. J Biomol NMR *54*, 267-283.

Figure S1

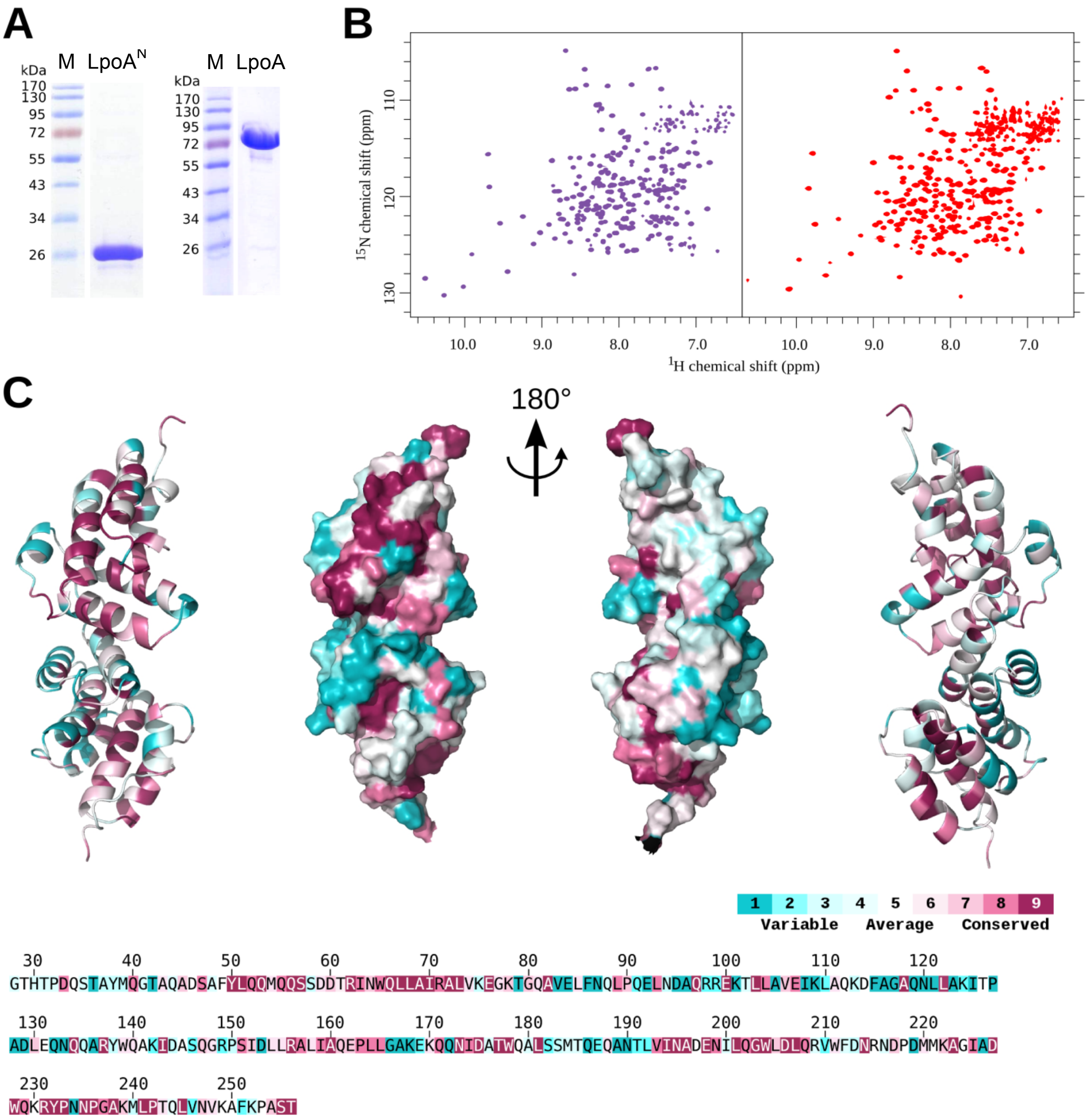

Figure S1, related to Figure 1: Characterization of LpoA^N and full-length LpoA in different conditions. (A) SDS-PAGE analysis of purified LpoA^N and LpoA. The purified proteins were separated by SDS-PAGE before the gels were stained with Coomassie Blue. The molecular weight of the protein markers shown in lanes M is listed. (B) The stability of LpoA^N in different temperature and pH conditions was evaluated by NMR. ^{1}H-^{15}N-BEST-TROSY spectrum recorded at 50°C and pH 4.5 in 150 mM sodium acetate buffer is shown in the left panel (purple). The equivalent spectrum recorded at 25°C and pH 7.0 after dialysis of the same LpoA^N sample in 20 mM HEPES buffer is shown in the right panel (red). Additional conditions (10 mM TRIS buffer at pH 7.5 and 25°C, 20 mM HEPES buffer containing 100 mM NaCl at pH 7.0 and 25°C, 100 mM

MOPS at pH 6.9 and 25°C, 100 mM MES buffer at pH 6.3 and 35°C, 200 mM sodium acetate buffer at pH 4.5 and 35°C) were investigated (data not shown). $LpoA^N$ was stable from 5 to 50°C and on the [4.5 – 7.5] pH range, as emphasized from the characteristic NMR peak pattern. The signal-to-noise ratio in the NMR spectra is increased at elevated temperature and acidic pH. (C) Conserved residues in $LpoA^N$ identified by the Consurf server (Ashkenazy et al., 2010). Multiple sequence alignment was built for $LpoA^N$ (residues 28 to 256) from 63 unique protein sequences using MAFFT and sequences from homologues were obtained from the UNIREF90 database and the CSI-BLAST algorithm. Conservation scores obtained from this alignment were used to color residues on a cartoon and a surface representation of the $LpoA^N$ structure. Two orientations rotated by 180° are shown for the two types of representations. On the bottom part of this panel, the conservation scores are reported on the primary sequence of the $LpoA^N$ protein.

Figure S2

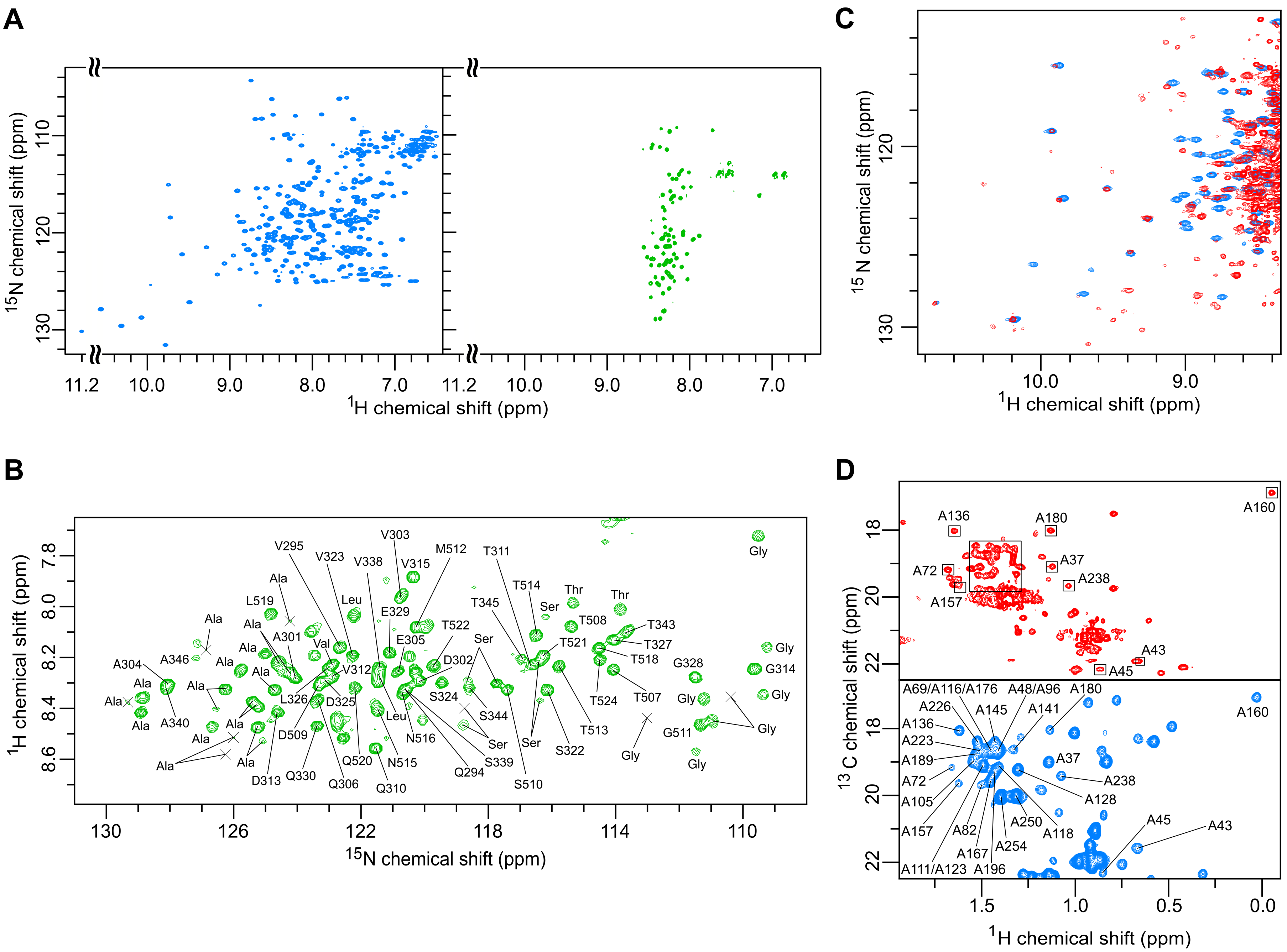
A
B
C
D
^{15}N chemical shift (ppm)
^{1}H chemical shift (ppm)
^{13}C chemical shift (ppm)
11.2
10.0
9.0
8.0
7.0
110
120
130
7.8
8.0
8.2
8.4
8.6
126
122
118
114
18
20
22
1.5
1.0
0.5
0.0
V303
V295
V323
V338
V315
M512
T311
T514
Thr
Ala
L519
Leu
E329
T345
Ser
T508
T343
T327
T518
T522
E305
T521
Ala
A301
Val
D302
G328
Gly
G314
A304
A346
V312
T524
T507
L326
D325
S324
S344
Ala
A340
D509
Q520
Q330
Q306
D313
Q310
N515
N516
Q294
S339
S510
S322
T513
G511
A136
A180
A160
A37
A72
A238
A157
A43
A45
A69/A116/A176
A48/A96
A226
A145
A141
A223
A189
A105
A82
A250
A118
A128
A167
A254
A111/A123
A196

Figure S2, related to Figure 2. Characteristic spectroscopic signatures of LpoA^{N} and full-length LpoA. (A) The ^{1}H-^{15}N-HSQC spectrum (left, blue) on LpoA^{N} was recorded at 50°C and pH 4.5 in a 100 mM sodium acetate buffer. The ^{1}H-^{15}N-BEST-TROSY spectrum (right, green) on ^{13}C,^{15}N-LpoA was recorded at 20°C and pH 6.5 in a 50 mM HEPES, 100 mM NaCl buffer. In this spectrum, only the amide resonances of residues located in flexible portions (mainly additional regions 1 and 2) were detected. (B) Portion of the LpoA spectrum shown in the previous right panel after rotation of the axes. Assignment of the resonances of disordered residues in LpoA was pursued with the collection of 3D HNCACB and BEST-TROSY-(H)N(COCA)NH. Residues sequentially assigned are indicated with the amino acid numbering of the wild-type LpoA protein. For the residues not sequentially assigned, the residue type obtained from the Cα and Cβ chemical shifts (for 36 resonances among which 19 Ala, which fits reasonably well with the 14 unassigned Ala in region 1 and the 2 Ala in region 2) is indicated by the name of the corresponding amino acid. Peaks observable only at lower threshold are marked with a cross. When the protein sample was stored for a few weeks at 4°C, the full-length ^{13}C,^{15}N-LpoA sample showed degradation of the C-terminal domain (as confirmed by gel electrophoresis and mass spectrometry) and reappearance of the signature of LpoA^{N} . At 20°C, the sample showed significant degradation within 48 h. (C) The left panel shows an overlay of the resolved portion of the 2D ^{1}H-^{15}N-BEST-TROSY (red) and ^{1}H-^{15}N-HSQC (blue) amide region collected on 72 μM U-[^{2}H, ^{12}C, ^{15}N], Val-[^{13}C^{1}H$_3$]$^{pro\text{-}S}$, Ala-[^{13}C^{1}H$_3$]-LpoA and 200 μM ^{13}C,^{15}N- LpoA^{N} samples, respectively, in 50 mM HEPES, 100 mM NaCl containing 10% D_2O at pH 6.5 and 293 K. This spectrum readily allows the identification of the N-terminal domain resonances in the full-length LpoA sample. ^{1}H-^{15}N correlations exclusively present in the full-length LpoA sample arise from the folded C-terminal domain for the main part, while missing correlations may arise from the partial reprotonation of amide positions as the sample was produced in a D_2O medium. (D) The top spectrum (red) shows a portion of the ^{1}H-^{13}C-methyl selective HMQC collected on the U-[^{2}H, ^{12}C, ^{15}N], Val-[^{13}C^{1}H$_3$]$^{pro\text{-}S}$, Ala-[^{13}C^{1}H$_3$]-LpoA sample in 50 mM HEPES, 100 mM NaCl containing 10% D_2O at pH 6.5 at 293K. Assignments of 27 out of

the 29 resonances of the N-terminal domain of LpoA could be transferred from the bottom spectrum (blue). This spectrum corresponds to a portion of the ^{1}H-^{13}C-CT-HSQC recorded at 50°C and pH 4.5 on the $LpoA^{N}$ sample in a 100 mM sodium acetate buffer. In the latter spectrum only Ala β resonances are labeled; Ile γ2, Val γ1 and γ2, and Thr γ resonances are kept unlabeled for clarity.

Figure S3

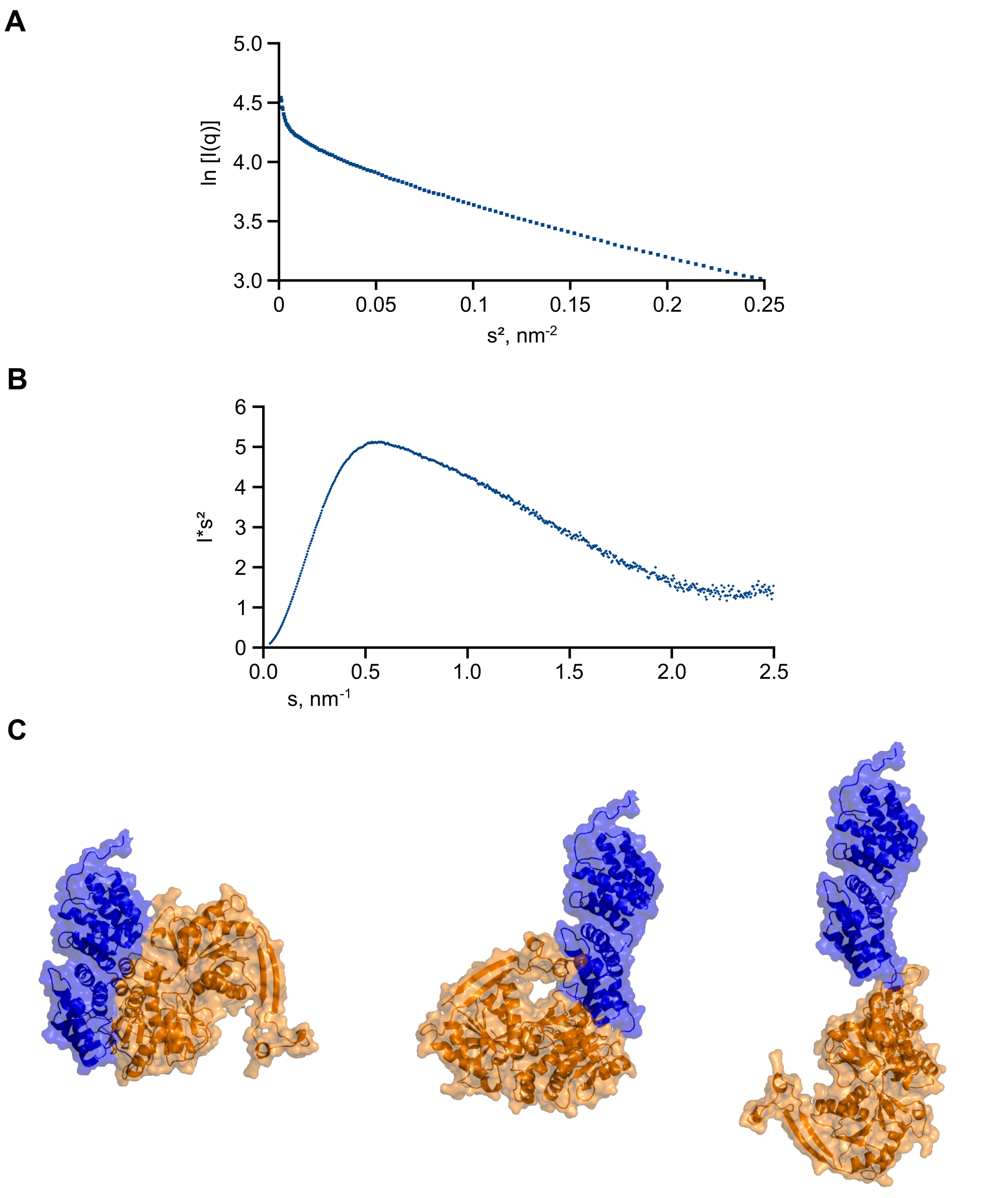

Figure S3, related to Figure 3. Complementary analysis of SAXS data collected on a 5 mg/mL LpoA sample. (A) The Guinier plot calculated from these data is consistent with a 73.4 kDa monomeric protein. (B) The Kratky profile is consistent with a partially unfolded protein, consistent

with AUC and NMR data discussed in the main text. (C) Ribbon representation of the globular (left), L-shaped (middle) and elongated (right) molecular models of LpoA (used to calculate the P(r) curves shown in Figure 3B). These models were built from the *E. coli* $LpoA^N$ NMR structure (blue) and the *E. coli* $LpoA^C$ structure (orange, modeled from the crystal structure of *H. influenzae* $LpoA^C$ (PDB code 3CKM) with PHYRE) by using different dihedral angles in the linker (Figure 2D).
